# Upfront Next Generation Sequencing in Non-Small Cell Lung Cancer

**DOI:** 10.3390/curroncol29070352

**Published:** 2022-06-22

**Authors:** Shelley Kuang, Andrea S. Fung, Kirstin A. Perdrizet, Kaitlin Chen, Janice J. N. Li, Lisa W. Le, Michael Cabanero, Ola Abu Al Karsaneh, Ming S. Tsao, Josh Morganstein, Laura Ranich, Adam C. Smith, Cuihong Wei, Carol Cheung, Frances A. Shepherd, Geoffrey Liu, Penelope Bradbury, Prodipto Pal, Joerg Schwock, Adrian G. Sacher, Jennifer H. Law, Tracy L. Stockley, Natasha B. Leighl

**Affiliations:** 1 Department of Medical Oncology, Princess Margaret Cancer Centre, University Health Network, University of Toronto, Toronto, ON M5G 2M9, Canada; shelley.kuang@uhn.ca (S.K.); andrea.fung@kingstonhsc.ca (A.S.F.); kirstin.perdrizet@williamoslerhs.ca (K.A.P.); k8lin.chen@gmail.com (K.C.); janice.li@uhn.ca (J.J.N.L.); frances.shepherd@uhn.ca (F.A.S.); geoffrey.liu@uhn.ca (G.L.); penelope.bradbury@uhn.ca (P.B.); adrian.sacher@uhn.ca (A.G.S.); jennifer.law@uhn.ca (J.H.L.); 2 Department of Biostatistics, Princess Margaret Cancer Centre, University Health Network, University of Toronto, Toronto, ON M5G 2M9, Canada; lisa.le@uhnresearch.ca; 3 Department of Laboratory Medicine & Pathology, Princess Margaret Cancer Centre, University Health Network, University of Toronto, Toronto, ON M5G 2M9, Canada; michael.cabanero@uhn.ca (M.C.); olakarasneh@yahoo.com (O.A.A.K.); ming.tsao@uhn.ca (M.S.T.); josh.morganstein7@gmail.com (J.M.); laura.ranich@uhn.ca (L.R.); adam.smith@uhn.ca (A.C.S.); cuihong.wei@uhn.ca (C.W.); carol.cheung@uhn.ca (C.C.); prodipto.pal@uhn.ca (P.P.); joerg.schwock@uhn.ca (J.S.); tracy.stockley@uhn.ca (T.L.S.); 4 Department of Basic Medical Sciences, Faculty of Medicine, The Hashemite University, Zarqa 13133, Jordan

**Keywords:** lung cancer, next generation sequencing, genomic alterations, Canada

## Abstract

In advanced non-small cell lung cancer (NSCLC), patients with actionable genomic alterations may derive additional clinical benefit from targeted treatment compared to cytotoxic chemotherapy. Current guidelines recommend extensive testing with next generation sequencing (NGS) panels. We investigated the impact of using a targeted NGS panel (TruSight Tumor 15, Illumina) as reflex testing for NSCLC samples at a single institution. Molecular analysis examined 15 genes for hotspot mutation variants, including *AKT1*, *BRAF*, *EGFR*, *ERBB2*, *FOXL2*, *GNA11*, *GNAQ*, *KIT*, *KRAS*, *MET*, *NRAS*, *PDGFRA*, *PIK3CA*, *RET* and *TP53* genes. Between February 2017 and October 2020, 1460 samples from 1395 patients were analyzed. 1201 patients (86.1%) had at least one variant identified, most frequently *TP53* (47.5%), *KRAS* (32.2%) or *EGFR* (24.2%). Among these, 994 patients (71.3%) had clinically relevant variants eligible for treatment with approved therapies or clinical trial enrollment. The incremental cost of NGS beyond single gene testing (*EGFR*, *ALK*) was CAD $233 per case. Reflex upfront NGS identified at least one actionable variant in more than 70% of patients with NSCLC, with minimal increase in testing cost. Implementation of NGS panels remains essential as treatment paradigms continue to evolve.

## 1. Introduction

In advanced non-small cell lung cancer (NSCLC), novel targeted treatment options continue to emerge as more oncogenic driver alterations are identified. Updated guidelines from the American Society of Clinical Oncology-Ontario Health (ASCO-OH), National Comprehensive Cancer Network (NCCN), International Association for the Study of Lung Cancer/College of American Pathology (IASLC/CAP) and European Society of Medical Oncology (ESMO) all recommend extensive testing with next generation sequencing (NGS) platforms to identify actionable alterations in *EGFR*, *ALK*, *ROS1*, *BRAF*, *HER2*, *KRAS*, *MET*, *NTRK* and *RET*, as well as immunochemistry for PD-L1 [1,2,3,4].

If actionable genomic alterations are identified, patients may gain access to targeted treatment options, which can improve patient outcomes including response, quality of life, progression-free survival and potentially overall survival compared to cytotoxic chemotherapy. Thus, the implementation of broader NGS platforms has become essential in the routine diagnosis and management of NSCLC patients. In managed care systems, assessment of the costs of broader testing and impact on patient care are also needed.

We investigated the impact of using a targeted NGS 15-gene panel (TruSight Tumor 15 [TST15], Illumina, San Diego, CA, USA) as part of the routine reflex testing for non-squamous NSCLC samples at a single institution.

## 2. Materials and Methods

The conduct of this prospective study was approved by the University Health Network (UHN) Research Ethics Board. Between February 2017 and October 2020, the UHN Genome Diagnostics Laboratory used the TST15 gene panel to test diagnostic samples of non-squamous NSCLC tumor tissue with reflex ordering by UHN pathologists, or as requested by the patient’s medical oncologist. Formalin fixed, paraffin embedded (FFPE) tumor samples were assessed for sufficiency and tumor rich areas identified by a board-certified pathologist, with a minimum tumor tissue surface area of 10 mm^2^ and ≥30% nucleated tumor cells required. DNA was extracted from macrodissected FFPE tissue. Molecular analysis used 20 ng DNA with a commercially available NGS targeted panel (TruSight Tumor 15, TST15, Illumina) sequenced on the MiSeq platform (2 × 150 bp configuration, Illumina). The TST15 includes regions of 15 genes covering hotspot variants, including single nucleotide variants and small insertions/deletions in the *AKT1*, *BRAF*, *EGFR*, *ERBB2*, *FOXL2*, *GNA11*, *GNAQ*, *KIT*, *KRAS*, *MET*, *NRAS*, *PDGFRA*, *PIK3CA*, *RET* and *TP53* genes. Bioinformatic analysis used MiSeq Reporter with manufacturer supplied TST analysis module (Illumina). Variants were classified according to Sukhai et al. [5]. In addition, samples underwent reflex testing for *ALK* gene fusions (5A4 IHC), PD-L1 (22C3 IHC pharmDx Assay) and in 2020, screening for *ROS1* fusions was initiated using IHC (D4D6 antibody) with FISH confirmation of positive cases [6,7,8].

Baseline demographic and treatment data were recorded prospectively including age, sex, smoking status, stage at diagnosis and pathologic subtype. For each specimen tested, the type of sample and site of origin were identified. Turnaround time (TAT) for profiling results was calculated from date of sample collection to report of genomic results to the oncologist, which included sample processing (formalin fixation and paraffin embedding), pathology review, clinical scientist review and final pathology sign-out. Molecular testing data were recorded based on the TST15 results as well as immunohistochemistry results for *ALK*, *ROS1* and PD-L1 expression. For patients who had multiple tumours tested, synchronous tumours were defined by repeat testing within 6 months, and metachronous tumours if testing was repeated more than 6 months later. Treatment and outcomes were collected manually and with automated natural language processing (DarwenTM), previously validated and shown to be highly accurate [9].

Actionable alterations were defined as variants which could be targetable using approved or active investigational therapies. Clinical trial eligibility was determined by the presence of interventional studies in NSCLC patients for the variant of interest, using ClinicalTrials.gov (see Data S1 for search terms). Incremental testing costs were calculated based on direct laboratory costs, including reagents, informatics, annotation, and technical time, but excluded overhead and administrative costs. Government reimbursement for single gene testing was subtracted. It was assumed that pathologist and pathology technician costs were similar whether TST15 or single gene (*EGFR*, EGFR-RT52, Entrogen, Woodland Hills, CA, USA) testing was used, as the pathology activities were not different for these tests.

## 3. Results

**Figure 1:**
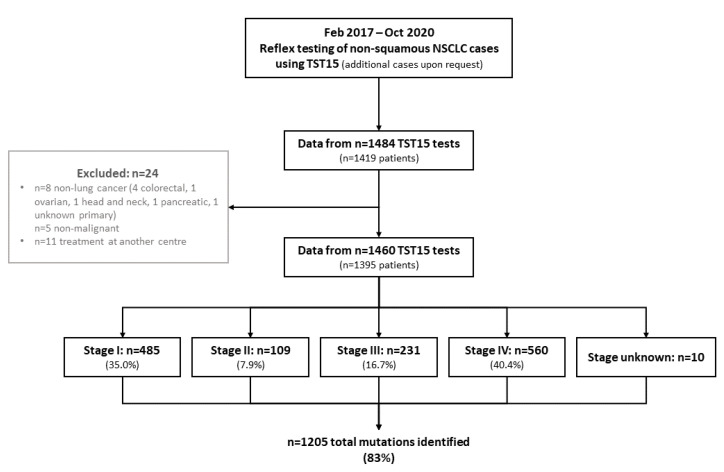
Study flow diagram.

Between February 2017 and October 2020, 1460 samples from 1395 patients were analyzed, with another 24 patient samples excluded due to non-lung cancer diagnosis or loss to follow up (Figure 1).

Baseline characteristics of patients and samples are listed in Table 1 and Table 2, respectively.

The median age of patients was 68.6 years, 52.3% were female, 33.1% were lifetime never smokers, and 85.9% had adenocarcinoma. Of the 1460 samples analyzed, 68.3% of samples were obtained from the lung cancer primary site; 45.5% of samples tested were from core biopsies. The mean turnaround time for reflex profiling results was 28.9 days (SD 8.9).

Of 1395 patients, 1201 patients (86.1%) had at least one variant identified in their cancer sample using TST15, while 405 (29.0%) had two or more co-mutations identified. The most frequently identified variants were in *TP53* (47.5%), followed by *KRAS* (32.2%) and *EGFR* (24.2%) (Table 3, full list in Data S2). Immunohistochemistry testing also identified 49 patients (4.1%) with tumor *ALK* fusions among 1202 patients who underwent testing, and 16 patients (1.1%) had *ROS1* rearrangements confirmed by FISH. PD-L1 TPS results were ≥50% for 337 patients (24.2%), 1–49% for 374 (26.8%) and negative (<1%) for 515 (36.9%). PD-L1 expression was unknown or testing inconclusive for 169 patients (12.1%).

Although most patients had single tumor sample sent for TST15 testing, 53 patients (3.8%) had multiple samples tested. Among these, 38 patients had synchronous samples tested and results were discordant for 20 (53%). For the 15 patients with metachronous tumor samples tested, 11 (73%) had discordant results.

When assessed based on smoking status, 444 patients were identified as never smokers, and 897 patients were former or current smokers. Among those who were never smokers, the most frequently exhibited variants were in *EGFR* (51.4%) and *TP53* (37.8%), while in previous and current smokers, variants in *TP53* (52.3%) and *KRAS* (43.8%) were more prevalent (Table 3).

When stratified by age, patients over and under 65 years of age demonstrated a similar frequency of tumor variants (Table 4).

**Figure 2:**
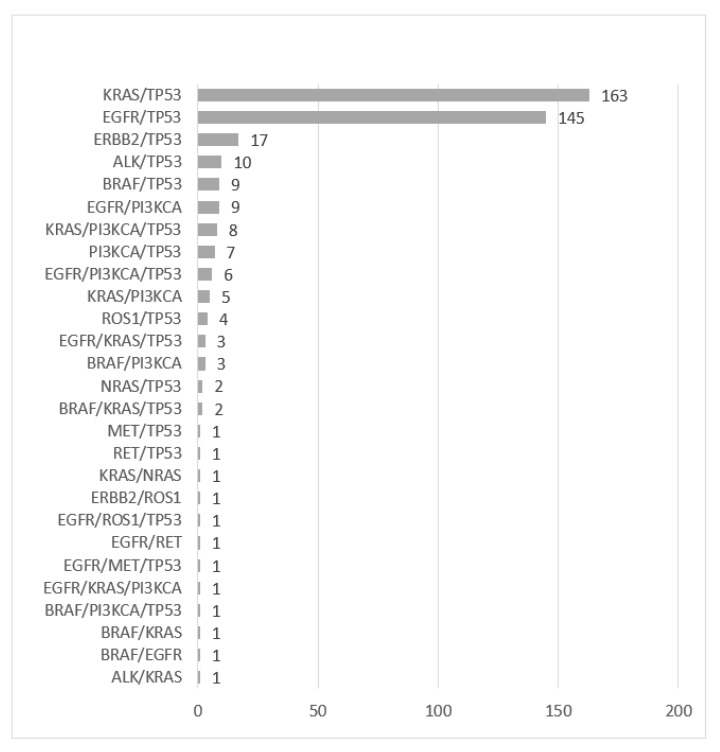
Genomic co-alterations identified (N = 1395 patients).

The most common co-mutations identified were in *KRAS/TP53* (163, 40.2%), followed by *EGFR/TP53* (145, 35.8%), *ERBB2/TP53* (17, 4.2%) and *BRAF/TP53* (9, 2.2%) (Figure 2).

**Figure 3:**
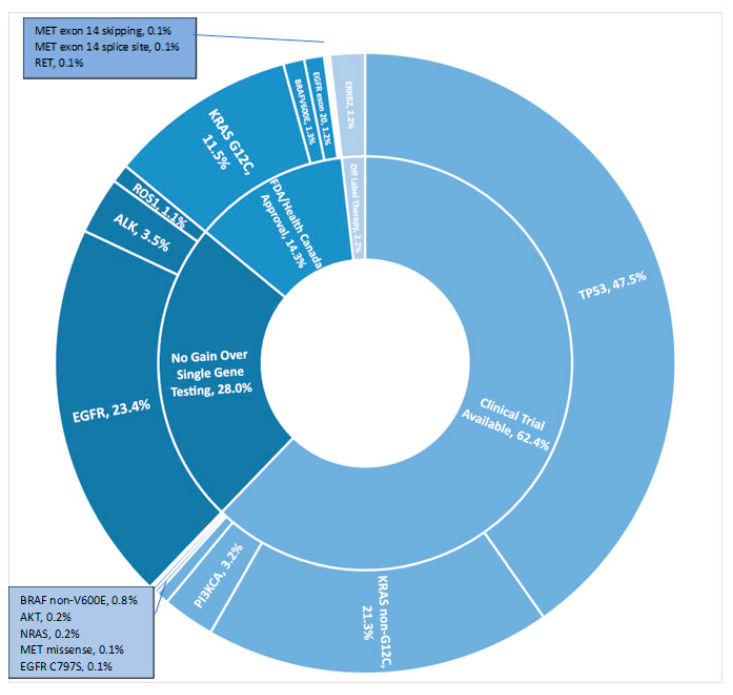
Incremental clinical benefit from use of 15-gene panel versus single gene testing.

Using TST15, clinically relevant variants were identified for 994 patients (71.3%), including 200 (14.3%) with Health Canada approved therapies, 870 (62.4%) for clinical trial enrolment (www.clinicaltrials.gov, accessed on 13 May 2022), and 30 (2.2%) for off-label treatments (e.g., afatinib or TDM-1 for *ERBB2* variants) (Figure 3).

The incremental cost of TST15 beyond reimbursed single gene testing for *EGFR* and *ALK* was CAD $233 per case.

In patients with advanced NSCLC, 203 received targeted therapy during their treatment course. Among them, 80 patients received 2 or more lines of targeted treatment for variants in *ERBB2*, *EGFR* (classic and exon 20 insertions), *KRAS G12C* and *MET*, as well as for *ALK* and *ROS1* rearrangements.

## 4. Discussion

Reflex upfront next generation sequencing with a 15-gene panel identified at least one variant in more than 80% of tested samples among patients with newly diagnosed non-small cell lung cancer. Of these, 71% derived incremental gain from testing by obtaining access to targeted therapy or becoming eligible for clinical trials based on genomic results, with only a minimal increase in testing costs (CAD $233 per case).

Advances in targeted therapies have led to updated NSCLC treatment guidelines across many gene variants. In metastatic NSCLC, treatment with targeted therapy may result in improved response rate, quality of life, progression-free and overall survival compared to cytotoxic chemotherapy in the first and subsequent line settings. In early stage NSCLC, the role of targeted therapy in the adjuvant setting continues to be explored in clinical trials. In addition to its therapeutic implications, the identification of oncogenic variants also provides valuable prognostic and predictive information [10].

*EGFR* variants were identified in 50% of never smokers and 10% of former and current smokers, consistent with its incidence in previously reported studies [11]. In contrast, *KRAS* variants were present in 43% of former and current smokers, which may be slightly higher than prior reports of 25–35% [12]. This could be related to the improved rate of variant detection using TST15 compared to previous methods of sequencing, as demonstrated in its validation study [13].

Although prior literature has described increased rate of molecular alterations in patients under 40 years old, this was not appreciated in this study, given the small sample size of patients within this age group [14]. Patients between the ages of 41–65 and over 65 had similar frequency of alterations.

For patients who had multiple samples tested, 53% of synchronous samples tested had discordant results, compared to 73% of metachronous samples tested. The reasons for this rate of discordance are unclear, and may be related to sampling heterogeneity and potentially increased diversity in later stages of tumor progression, related to the evolution of subclones [15].

One limitation of the NGS testing in our study was the turnaround time between sample collection and report of results, with a mean of 29 days. This time included multiple steps in the process, including sample pathology processing (formalin fixation and paraffin embedding), pathology review, laboratory NGS testing, clinical scientist review and final pathology sign-out. In addition, the TST15 panel was run in batches for cost reasons. However, current Ontario provincial guidelines recommend that NGS testing be completed within 14 days of sample collection, indicating that there is a need for more resources dedicated to improving TAT [16].

However, even the proposed turnaround times may still be too long for some patients. We have shown previously that only 21% of patients have molecular testing results available at the time of initial medical oncology consultation. Furthermore, delayed results led to delayed initiation of treatment, and 19% of patients eligible for targeted therapy received chemotherapy instead [17]. This would have implications for patients with high PD-L1 expression, who are at risk of starting immunotherapy instead of targeted therapy if genomic results are unavailable, recognizing that checkpoint inhibitor monotherapy in prior studies has been less effective in patients with oncogenic driver alterations. If these patients were switched to targeted therapy in the future, this may also increase their risk of important treatment-related adverse events, such as pneumonitis and hepatitis [18].

Sample quantity and quality remain key issues for successful testing [19,20,21], and NGS can provide a simultaneous result on multiple genes, thus avoiding use of tissue in multiple sequential tests. However, in cases where tissue is very small, immunohistochemistry remains an important modality in rapid assessment of *ALK* and *ROS1* rearrangements and PD-L1 expression in patients with NSCLC [6,7,8,22]. IHC may also be advantageous over NGS in cases when a shorter turnaround time is required, and for the detection of fusion genes such as *NTRK*. The identification of oncogenic protein expression through IHC may also be predictive for response to targeted therapy [23].

While the use of NGS rapidly expands the population eligible for targeted therapy, many patients may have challenges in accessing these novel agents due to high cost, particularly those in publicly funded systems or without private drug insurance [24]. As diagnostic and therapeutic advancements continue in the field of thoracic oncology, identification of genomic alterations is pivotal in our ability to gain access to novel therapies that improve patient and system outcomes. Current guidelines recommend comprehensive assessment of multiple variant types, including single nucleotide variants, small insertions/deletions, fusions and copy number variations [25]. Given the impact on clinical outcomes, development of comprehensive and affordable NGS panels is essential as standard of care molecular testing requirements continue to evolve.

## Figures and Tables

**Table 1:** Patient and disease characteristics (N = 1395 patients).

	Number (%)
	N = 1395
**Age at diagnosis, median** (range)	68.6 years (18.8–97.2)
**Sex**	
Female	730 (52.3%)
Male	665 (47.7%)
**Smoking status**	
Never	444 (33.1%)
Former Smoker	492 (36.7%)
Current Smoker	405 (30.2%)
Unknown	54
**Stage at diagnosis**	
I	485 (35.0%)
II	109 (7.9%)
III	231 (16.7%)
IV	560 (40.4%)
Unknown	10
**Histology**	
Adenocarcinoma	1198 (85.9%)
Large Cell	40 (2.9%)
Squamous	34 (2.4%)
Pleomorphic/Sarcomatoid	14 (1.0%)
Small Cell	12 (0.9%)
Mixed histology	6 (1.2%)
Not otherwise specified	91 (6.5%)

**Table 2:** Sample characteristics (N = 1460 samples).

	Number (%)
	N = 1460
Samples tested per patient *	
1	1335 (95.7%)
2	55 (3.9%)
3	5 (0.4%)
**Sample type**	
Core biopsy	665 (45.5%)
Surgical specimen	379 (26.0%)
FNA cytology	353 (24.2%)
Exfoliative cytology	62 (4.2%)
Unknown	1
**Sample site**	
Primary (lung)	997 (68.3%)
Non-bone visceral or soft tissue metastasis	357 (24.5%)
Pleural fluid	57 (3.9%)
Bone metastasis	33 (2.3%)
Other	16 (1.1%)

* 7 of 60 patients with multiple samples tested had unsuccessful profiling of at least one sample.

**Table 3:** Molecular results based on smoking status.

Gene Variant	Never Smoker (N = 444)	Former/Current Smoker (N = 897)	All Patients (N = 1395)
**AKT**	**0**	**3 (0.3%)**	**3 (0.2%)**
**BRAF**	**7 (1.6%)**	**21 (2.3%)**	**29 (2.1%)**
*V600E* *	6	11	18
Non-*V600E*	1	10	11
**EGFR**	**228 (51.4%)**	**97 (10.8%)**	**337 (24.2%)**
Exon 19 deletion *	102	41	147
*L858R* *	98	34	137
Other Exon 19/20/21	12	12	27
Exon 18 *	13	10	24
Exon 20 insertion *	17	1	18
*T790M* *	11	15	16
*L861Q* *	5	5	10
*C797S*	1	0	1
≥2 EGFR variants	28	11	40
**ERBB2**	**20 (4.5%)**	**10 (1.1%)**	**30 (2.2%)**
Exon 20	17	5	22
Transmembrane domain	2	2	4
Other	1	3	4
**KRAS**	**40 (9.0%)**	**393 (43.8%)**	**449 (32.2%)**
*G12C* *	4	152	161
Non-*G12C*	36	253	300
≥2 *KRAS* variants	0	12	12
**MET**	**1 (0.1%)**	**3 (0.3%)**	**4 (0.3%)**
Exon 14 splice site *	1	1	2
Exon 14 skipping *	0	1	1
Non-splice site missense	0	1	1
**NRAS**	**1 (0.1%)**	**2 (0.2%)**	**3 (0.2%)**
**PI3KCA**	**15 (3.4%)**	**29 (3.2%)**	**45 (3.2%)**
RET *	**0**	**2 (0.2%)**	**2 (0.1%)**
**TP53**	**168 (37.8%)**	**469 (52.3%)**	**662 (47.5%)**

* Actionable alterations with approved targeted therapy.

**Table 4:** Molecular results by patient age.

Gene Variant	<40 years (N = 19)	40-65 years (N = 506)	≥65 years (N = 870)
**AKT**	**0**	**1 (0.2%)**	**2 (0.2%)**
**BRAF**	**0**	**6 (1.2%)**	**23 (2.6%)**
*V600E* *	0	5	13
Non-*V600E*	0	1	10
**EGFR**	**2 (10.5%)**	**132 (26.1%)**	**203 (23.3%)**
Exon 19 deletion *	0	68	79
*L858R* *	1	48	88
Other Exon 19/20/21	0	10	17
Exon 18 *	1	7	16
Exon 20 insertion *	0	7	11
*T790M* *	0	8	8
*L861Q* *	0	2	8
*C797S*	0	1	0
≥2 EGFR variants	0	17	23
**ERBB2**	**1 (5.3%)**	**14 (2.8%)**	**15 (1.7%)**
Exon 20	1	9	12
Transmembrane domain	0	2	2
Other	0	3	1
**KRAS**	**1 (5.3%)**	**153 (30.2%)**	**295 (33.9%)**
*G12C* *	0	55	106
Non-*G12C*	1	102	197
≥2 *KRAS* variants	0	4	8
**MET**	**0**	**0**	**4 (0.5%)**
Exon 14 splice site *	0	0	2
Exon 14 skipping *	0	0	1
Non-splice site missense	0	0	1
**NRAS**	**0**	**0**	**3 (0.3%)**
**PI3KCA**	**0**	**13 (2.6%)**	**32 (3.7%)**
RET *	**0**	**2 (0.4%)**	**0**
**TP53**	**9 (47.4%)**	**251 (49.6%)**	**402 (46.2%)**

* Actionable alterations with approved targeted therapy.

## Data Availability

The data presented in this study is available in this article (and supplementary material).

## Supplementary Materials

The following are available online at https://www.mdpi.com/article/10.3390/curroncol29070352/s1, Data S1. Search terms used on ClinicalTrials.gov; Data S2. Variants detected using TST15.
