# Supplementary material for: Upfront Next Generation Sequencing in Non-Small Cell Lung Cancer
Source: Curr Oncol. 2022 Jun 22;29(7):4428–37. doi: 10.3390/curroncol29070352 (PMC9319994; doi:10.3390/curroncol29070352)
Supplement: Supplementary file 1 [file curroncol-29-00352-s001.zip › LUNGS1 16 june 2022 supplemental 1 clintrials.pdf]

Supplementary Data S1. Search terms used on ClinicalTrials.gov.

Search
Condition or disease
-Lung Neoplasm
AND
-Advanced Neoplasms
AND
-Thoracic Neoplasm

Other
-Gene name (*AKT1, BRAF, ERBB2, FOXL2, GNA11, GNAQ, KIT, KRAS, MET, NRAS, PDGFRA, PIK3CA, RET, TP53*)

Study Type
All

Study Results
All

Status Recruitment
-Recruiting
OR
-Enrolling by invitation

Eligibility Criteria
Age
-Adult (18-64)
AND
-Older Adult (65+)

Sex
-All

Location
-All
