# Supplementary material for: Upfront Next Generation Sequencing in Non-Small Cell Lung Cancer
Source: Curr Oncol. 2022 Jun 22;29(7):4428–37. doi: 10.3390/curroncol29070352 (PMC9319994; doi:10.3390/curroncol29070352)
Supplement: Supplementary file 1 [file curroncol-29-00352-s001.zip › LUNGS1 16 june 2022 supplemental 2 variants no code.pdf]

Supplementary Data S2. Variants detected using TST15.

Gene Variant	Type	Alteration and VAF
AKT	GLU17LYS	c.49G>A (p.Glu17Lys) 25%
AKT	GLU17LYS	c.49G>A (p.Glu17Lys) 9%
AKT	GLU17LYS	c.49G>A (p.Glu17Lys) 16%
BRAF	NON-V600E	c.1742A>T (p.Asn581Ile) 8%
BRAF	NON-V600E	c.1756G>A (p.Glu586Lys) 12%
BRAF	NON-V600E	c.1786G>C (p.Gly596Arg) 47%
BRAF	NON-V600E	c.1756G>A (p.Glu586Lys) 58%
BRAF	NON-V600E	c.1798_1799delGTinsAA (p.Val600Lys) 8%
BRAF	NON-V600E	c.1781A>C (p.Asp594Ala) 12%
BRAF	NON-V600E	c.1803A>C (p.Lys601Asn) 39%
BRAF	NON-V600E	c.1790T>G (p.Leu597Arg) 42%
BRAF	NON-V600E	c.1801A>G (p.Lys601Glu) 14%
BRAF	NON-V600E	c.1803A>T (p.Lys601Asn)
BRAF	NON-V600E	c.1801A>G (p.Lys601Glu) 25%
BRAF	V600E	c.1799T>A (p.Val600Glu) 38%
BRAF	V600E	c.1799T>A (p.Val600Glu) 18%
BRAF	V600E	c.1799T>A (p.Val600Glu) 47%
BRAF	V600E	c.1799T>A (p.Val600Glu) 12%
BRAF	V600E	c.1799T>A (p.Val600Glu) 43%
BRAF	V600E	c.1799T>A (p.Val600Glu) 25%
BRAF	V600E	c.1799T>A (p.Val600Glu) 20%
BRAF	V600E	c.1799T>A (p.Val600Glu)
BRAF	V600E	c.1799T>A (p.Val600Glu) 27%
BRAF	V600E	c.1799T>A (p.Val600Glu) 27%
BRAF	V600E	c.1799T>A (p.Val600Glu) 15%
BRAF	V600E	c.1799T>A (p.Val600Glu) 20%
BRAF	V600E	c.1799T>A (p.Val600Glu) 11%
BRAF	V600E	c.1799T>A (p.Val600Glu)
BRAF	V600E	c.1799T>A (p.Val600Glu) 28%
BRAF	V600E	c.1799T>A (p.Val600Glu) 27%
BRAF	V600E	c.1799T>A (p.Val600Glu) 17%
BRAF	V600E	c.1799T>A (p.Val600Glu)
EGFR	C797S	c.2389T>A (p.Cys797Ser) 14%
EGFR	EXON 18	c.2156G>C (p.Gly719Ala) 20%
EGFR	EXON 18	c.2125G>A (p.Glu709Lys) 59%
EGFR	EXON 18	c.2155G>T (p.Gly719Cys)
EGFR	EXON 18	c.2156G>C (p.Gly719Ala)
EGFR	EXON 18	c.2155G>T (p.Gly719Cys)
EGFR	EXON 18	c.2156G>C (p.Gly719Ala) 5%
EGFR	EXON 18	c.2156G>C (p.Gly719Ala) 19%
EGFR	EXON 18	c.2156G>C (p.Gly719Ala) 33%
EGFR	EXON 18	c.2164G>T (p.Ala722Ser) 16%
EGFR	EXON 18	c.2155G>A (p.Gly719Ser)
EGFR	EXON 18	c.2155G>T (p.Gly719Cys) 34%

EGFR	EXON 18	c.2126A>C (p.Glu709Ala) 35%
EGFR	EXON 18	c.2121G>T (p.Leu707Phe) (31%)
EGFR	EXON 18	c.2170G>A (p.Gly724Ser)
EGFR	EXON 18	c.2156G>C (p.Gly719Ala) 7%
EGFR	EXON 18	c.2155G>A (p.Gly719Ser) 92%
EGFR	EXON 18	c.2126A>C (p. Glu709Ala) 92%
EGFR	EXON 18	c.2155G>A (p.Gly719Ser) 11%
EGFR	EXON 18	c.2065G>A (p.Val689Met) 52%
EGFR	EXON 18	c.2155G>A (p.Gly719Ser) 22%
EGFR	EXON 18	c.2156G>C (p.Gly719Ala) 48%
EGFR	EXON 18	c.2126A>C (p.Glu709Ala) 48%
EGFR	EXON 18	c.2159C>T (p.Ser720Phe) 16%
EGFR	EXON 18	c.2156G>C (p.Gly719Ala) 94%
EGFR	EXON 18	c.2126A>C (p.Glu709Ala) 94%
EGFR	EXON 18	c.2152C>G (p.Leu718Val) (9%)
EGFR	EXON 18	c.2156G>C (p.Gly719Ala) 62%
EGFR	EXON 19 DELETION	c.2235_2249delGGAATTAAGAGAAGC (p.Glu746_Ala750del) 76%
EGFR	EXON 19 DELETION	c.2237_2255delAATTAAGAGAAGCAACATCinsT (p.Glu746_Ser752delinsVal) 16%
EGFR	EXON 19 DELETION	c.2235_2249delGGAATTAAGAGAAGC (p.Glu746_Ala750del) 35%
EGFR	EXON 19 DELETION	c.2235_2249delGGAATTAAGAGAAGC (p.Glu746_Ala750del) 42%
EGFR	EXON 19 DELETION	c.2235_2249delGGAATTAAGAGAAGC (p.Glu746_Ala750del) 66%
EGFR	EXON 19 DELETION	c.2240_2257delTAAGAGAAGCAACATCTC (p.Leu747_Pro753delinsSer)
EGFR	EXON 19 DELETION	c.2240_2257delTAAGAGAAGCAACATCTC (p.Leu747_Pro753delinsSer) 74%
EGFR	EXON 19 DELETION	c.2236_2250delGAATTAAGAGAAGCA (p.Glu746_Ala750del) 19%
EGFR	EXON 19 DELETION	c.2240_2257delTAAGAGAAGCAACATCTC (p.Leu747_Pro753delinsSer)
EGFR	EXON 19 DELETION	c.2240_2254delTAAGAGAAGCAACAT (p.Leu747_Thr751del) 16%
EGFR	EXON 19 DELETION	c. 2236_2250delGAATTAAGAGAAGCA (p.Glu746_Ala750del) 15%
EGFR	EXON 19 DELETION	(p.Glu746_Ala750del) 8%
EGFR	EXON 19 DELETION	(p.Leu747_Pro753delinsSer) 14%
EGFR	EXON 19 DELETION	c.2236_2250delGAATTAAGAGAAGCA (p.Glu746_Ala750del) 49%
EGFR	EXON 19 DELETION	c.2235_2249delGGAATTAAGAGAAGC (p.Glu746_Ala750del) 21%
EGFR	EXON 19 DELETION	(p.Glu746_Ala750del) 15%
EGFR	EXON 19 DELETION	c.2239_2263delinsCCAACAT (p.Leu747_Ala755delinsProThrSer) 54%
EGFR	EXON 19 DELETION	c.2235_2249delGGAATTAAGAGAAGC (p.Glu746_Ala750del)
EGFR	EXON 19 DELETION	c.2239_2248delTTAAGAGAAGinsC (p.Leu747_Ala750delinsPro) 7%
EGFR	EXON 19 DELETION	c.2235_2249delGGAATTAAGAGAAGC (p.Glu746_Ala750del) 52%
EGFR	EXON 19 DELETION	c.2235_2249delGGAATTAAGAGAAGC (p.Glu746_Ala750del) 55%
EGFR	EXON 19 DELETION	c.2236_2250delGAATTAAGAGAAGCA (p.Glu746_Ala750del) 24%
EGFR	EXON 19 DELETION	(p.Glu746_Ala750del) 21%
EGFR	EXON 19 DELETION	(p.Glu746_Ala750del) 54%
EGFR	EXON 19 DELETION	(p.Leu747_Ala750delinsPro) 21%
EGFR	EXON 19 DELETION	c.2235_2249delGGAATTAAGAGAAGC (p.Glu746_Ala750del) 25%
EGFR	EXON 19 DELETION	c.2235_2249delGGAATTAAGAGAAGC (p.Glu746_Ala750del) 24%
EGFR	EXON 19 DELETION	(p.Glu746_Ala750del) 10%
EGFR	EXON 19 DELETION	(p.Glu746_Ala750del) 30%
EGFR	EXON 19 DELETION	c.2235_2249delGGAATTAAGAGAAGC (p.Glu746_Ala750del) 72%
EGFR	EXON 19 DELETION	(p.Glu746_Ala750del) 23%

EGFR	EXON 19 DELETION	c.2236_2250delGAATTAAGAGAAGCA (p.Glu746_Ala750del) 29%
EGFR	EXON 19 DELETION	c.2235_2249delGGAATTAAGAGAAGC (p.Glu746_Ala750del) 61%
EGFR	EXON 19 DELETION	c.2240_2254delTAAGAGAAGCAACAT (p.Leu747_Thr751del) 27%
EGFR	EXON 19 DELETION	(p.Glu746_Ala750del) 65%
EGFR	EXON 19 DELETION	(p.Leu747_Ala750delinsPro) 23%
EGFR	EXON 19 DELETION	(p.Glu746_Ala750del) 30%
EGFR	EXON 19 DELETION	(p.Glu746_Ala750del) 21%
EGFR	EXON 19 DELETION	c.2235_2249delGGAATTAAGAGAAGC (p.Glu746Ala750del) 23%
EGFR	EXON 19 DELETION	(p.Leu747_Thr751del) 75%
EGFR	EXON 19 DELETION	(p.Glu746_Ala750del) 43%
EGFR	EXON 19 DELETION	c.2237_2255delinsT (p.Glu746_Ser752delinsVal) 19%
EGFR	EXON 19 DELETION	c.2235_2249delGGAATTAAGAGAAGC (p.Glu746_Ala750del)
EGFR	EXON 19 DELETION	(p.Leu747_Ala750delinsPro) 24%
EGFR	EXON 19 DELETION	c.2236_2250delGAATTAAGAGAAGCA (p.Glu746_Ala750del) 38%
EGFR	EXON 19 DELETION	c.2237_2251delAATTAAGAGAAGCAA (p.Glu746_Thr751delinsAla) 54%
EGFR	EXON 19 DELETION	c.2235_2249delGGAATTAAGAGAAGC (p.Glu746_Ala750del) 40%
EGFR	EXON 19 DELETION	c.2235_2249delGGAATTAAGAGAAGC (p.Glu746_Ala750del)
EGFR	EXON 19 DELETION	c.2236_2250delGAATTAAGAGAAGCA (p.Glu746_Ala750del) 11%
EGFR	EXON 19 DELETION	c.2235_2249delGGAATTAAGAGAAGC (p.Glu746_Ala750del) 8%
EGFR	EXON 19 DELETION	c.2237_2255delAATTAAGAGAAGCAACATCinsT (p.Glu746_Ser752delinsVal) 37%
EGFR	EXON 19 DELETION	c.2236_2250delGAATTAAGAGAAGCA (p.Glu746_Ala750del) 28%
EGFR	EXON 19 DELETION	c.2236_2250delGAATTAAGAGAAGCA (p.Glu746_Ala750del) 86%
EGFR	EXON 19 DELETION	c.2236_2250delGAATTAAGAGAAGCA (p.Glu746_Ala750del) 11%
EGFR	EXON 19 DELETION	c.2236_2250delGAATTAAGAGAAGCA (p.Glu746_Ala750del) 36%
EGFR	EXON 19 DELETION	c.2236_2250delGAATTAAGAGAAGCA (p.Glu746_Ala750del) 14%
EGFR	EXON 19 DELETION	c.2235_2249delGGAATTAAGAGAAGC (p.Glu746_Ala750del)
EGFR	EXON 19 DELETION	c.2236_2250delGAATTAAGAGAAGCA (p.Glu746_Ala750del) 20%
EGFR	EXON 19 DELETION	c.2237_2255delAATTAAGAGAAGCAACATCinsT (p.Glu746_Ser752delinsVal)
EGFR	EXON 19 DELETION	c.2235_2249delGGAATTAAGAGAAGC (p.Glu746_Ala750del) 76%
EGFR	EXON 19 DELETION	c.2236_2250delGAATTAAGAGAAGCA (p.Glu746_Ala750del) 17%
EGFR	EXON 19 DELETION	c.2235_2249delGGAATTAAGAGAAGC (p.Glu746_Ala750del) 19%
EGFR	EXON 19 DELETION	c.2235_2249delGGAATTAAGAGAAGC (p.Glu746_Ala750del) 30%
EGFR	EXON 19 DELETION	c.2236_2250delGAATTAAGAGAAGCA (p.Glu746_Ala750del) 32%
EGFR	EXON 19 DELETION	c.2235_2249delGGAATTAAGAGAAGC (p.Glu746_Ala750del) 25%
EGFR	EXON 19 DELETION	c.2240_2254delTAAGAGAAGCAACAT (p.Leu747_Thr751del) 81%
EGFR	EXON 19 DELETION	c.2240_2254delTAAGAGAAGCAACAT (p.Leu747_Thr751del) 38%
EGFR	EXON 19 DELETION	c.2237_2257del (p.Glu746_Pro753delinsAla) 18%
EGFR	EXON 19 DELETION	c.2236_2250delGAATTAAGAGAAGCA (p.Glu746_Ala750del) 34%
EGFR	EXON 19 DELETION	c.2235_2249delGGAATTAAGAGAAGC (p.Glu746_Ala750del) 15%
EGFR	EXON 19 DELETION	c.2235_2249delGGAATTAAGAGAAGC (p.Glu746_Ala750del) 92%
EGFR	EXON 19 DELETION	c.2255C>G (p.Ser752Cys) 16%
EGFR	EXON 19 DELETION	c.2235_2249delGGAATTAAGAGAAGC (p.Glu746_Ala750del) 48%
EGFR	EXON 19 DELETION	c.2235_2249delGGAATTAAGAGAAGC (p.Glu746_Ala750del) 14%
EGFR	EXON 19 DELETION	c.2236_2250delGAATTAAGAGAAGCA (p.Glu746_Ala750del) 55%
EGFR	EXON 19 DELETION	c.2240_2257delTAAGAGAAGCAACATCTA (p. leu747Pro753delinsSer) 54%
EGFR	EXON 19 DELETION	c.2237_2251delinsTTC(p.Glu746_Thr751delinsAla) 55%
EGFR	EXON 19 DELETION	c.2235_2249delGGAATTAAGAGAAGC (p.Glu746_Ala750del) 46%

EGFR	EXON 19 DELETION	c.2239_2248delTTAAGAGAAGinsC (p.Leu747_Ala750delinsPro) 70%
EGFR	EXON 19 DELETION	c.2237_2255delAATTAAGAGAAGCAACATCinsT (p.Glu746_Ser752delinsVal) 48%
EGFR	EXON 19 DELETION	c.2237_2255delAATTAAGAGAAGCAACATCinsT (p.Glu746_Ser752delinsVal) 57%
EGFR	EXON 19 DELETION	c.2236_2250delGAATTAAGAGAAGCA (p. Glu746_Ala750del) 38%
EGFR	EXON 19 DELETION	c.2240_2254delTAAGAGAAGCAACAT (p.Leu747_Thr751del) 30%
EGFR	EXON 19 DELETION	c.2240_2254delTAAGAGAAGCAACAT (p.Leu747_Thr751del) 76%
EGFR	EXON 19 DELETION	c.2236_2250del (p.Glu746_Ala750del)
EGFR	EXON 19 DELETION	c.2236_2250delGAATTAAGAGAAGCA (p.Glu746_Ala750del) 8%
EGFR	EXON 19 DELETION	c.2235_2249delGGAATTAAGAGAAGC (p.Glu746_Ala750del) 9%
EGFR	EXON 19 DELETION	c.2236_2250delGAATTAAGAGAAGCA (p.Glu746_Ala750del) 17%
EGFR	EXON 19 DELETION	c.2239_2256delTTAAGAGAAGCAACATCT (p.Leu747_Ser752del)
EGFR	EXON 19 DELETION	c.2236_2250delGAATTAAGAGAAGCA (p. Glu746_Ala750del) 63%
EGFR	EXON 19 DELETION	c.2235_2249delGGAATTAAGAGAAGC (p.Glu746_Ala750del) 23%
EGFR	EXON 19 DELETION	c.2236_2250delGAATTAAGAGAAGCA (p. Glu746_Ala750del) 24%
EGFR	EXON 19 DELETION	c.2236_2250delGAATTAAGAGAAGCA (p. Glu746_Ala750del) 32%
EGFR	EXON 19 DELETION	c.2240_2257delTAAGAGAAGCAACATCTC (p. Leu747_Pro753delinsSer) 48%
EGFR	EXON 19 DELETION	c.2235_2249delGGAATTAAGAGAAGC (p. Glu746_Ala750del) 44%
EGFR	EXON 19 DELETION	c.2235_2249delGGAATTAAGAGAAGC (p. Glu746_Ala750del) 50%
EGFR	EXON 19 DELETION	c.2236_2250delGAATTAAGAGAAGCA (p.Glu746_Ala750del) 16%
EGFR	EXON 19 DELETION	c.2239_2248delTTAAGAGAAGinsC (p.Leu747_Ala750delinsPro) 24%
EGFR	EXON 19 DELETION	c.2235_2249delGGAATTAAGAGAAGC (p.GLU746_ALA750del) 47%
EGFR	EXON 19 DELETION	c.2237_2255delAATTAAGAGAAGCAACATCinsT (p.Glu746_Ser752delinsVal) 16%
EGFR	EXON 19 DELETION	c.2235_2240delinsAATTCC (p.Glu746_Leu747delinsIlePro) 32%
EGFR	EXON 19 DELETION	c.2240_2254delTAAGAGAAGCAACAT (p.Leu747_Thr751del) 84%
EGFR	EXON 19 DELETION	c.2235_2249delGGAATTAAGAGAAGC (p.Glu746_Ala750del)
EGFR	EXON 19 DELETION	Unknown
EGFR	EXON 19 DELETION	c.2239_2248delTTAAGAGAAGinsC (p.Leu747_Ala750delinsPro) 70%
EGFR	EXON 19 DELETION	c.2235_2249delGGAATTAAGAGAAGC (p.Glu746_Ala750del) 26%
EGFR	EXON 19 DELETION	c.2235_2252delGGAATTAAGAGAAGCAACinsAAT (p.Glu746_Thr751delinsIle) 31%
EGFR	EXON 19 DELETION	c.2235_2249delGGAATTAAGAGAAGC (p.Glu746_Ala750del) 49%
EGFR	EXON 19 DELETION	c.2235_2249delGGAATTAAGAGAAGC (p.Glu746_Ala750del)
EGFR	EXON 19 DELETION	c.2235_2249delGGAATTAAGAGAAGC (p.Glu746_Ala750del) 13%
EGFR	EXON 19 DELETION	c.2240_2254delTAAGAGAAGCAACAT (p.Leu747_Thr751del) 54%
EGFR	EXON 19 DELETION	c.2232_2249delinsAAA (p.Glu746_Ala750del) 10%
EGFR	EXON 19 DELETION	c.2235_2249delGGAATTAAGAGAAGC (p.Glu746_Ala750del) 18%
EGFR	EXON 19 DELETION	c.2236_2250delGAATTAAGAGAAGCA (p.Glu746_Ala750del) 58%
EGFR	EXON 19 DELETION	c.2236_2250delGAATTAAGAGAAGCA (p.Glu746_Ala750del) 49%
EGFR	EXON 19 DELETION	c.2235_2249delGGAATTAAGAGAAGC (p.Glu746_Ala750del) 64%
EGFR	EXON 19 DELETION	c.2235_2249delGGAATTAAGAGAAGC (p.Glu746_Ala750del) 90%
EGFR	EXON 19 DELETION	c.2235_2249delGGAATTAAGAGAAGC (p.Glu746_Ala750del) 11%
EGFR	EXON 19 DELETION	c.2235_2249delGGAATTAAGAGAAGC (p.Glu746_Ala750del) 38%
EGFR	EXON 19 DELETION	c.2235_2249delGGAATTAAGAGAAGC (p.Glu746_Ala750del) 28%
EGFR	EXON 19 DELETION	c.2236_2250delGAATTAAGAGAAGCA (p.Glu746_Ala750del) 17%
EGFR	EXON 19 DELETION	c.2239_2248delTTAAGAGAAGinsC (p.Leu747_Ala750delinsPro) 90%
EGFR	EXON 19 DELETION	c.2236_2250delGAATTAAGAGAAGCA (p.Glu746_Ala750del) 41%
EGFR	EXON 19 DELETION	c.2239_2256delTTAAGAGAAGCAACATCT (p.Leu747_Ser752del) 48%
EGFR	EXON 19 DELETION	c.2235_2249delGGAATTAAGAGAAGC (p.Glu746_Ala750del) 20%

EGFR	EXON 19 DELETION	c.2235_2249delGGAATTAAGAGAAGC (p.Glu746_Ala750del) 28%
EGFR	EXON 19 DELETION	c.2235_2249delGGAATTAAGAGAAGC (p.Glu746_Ala750del) 23%
EGFR	EXON 19 DELETION	c.2235_2249delGGAATTAAGAGAAGC (p.Glu746_Ala750del) 34%
EGFR	EXON 19 DELETION	c.2240_2257delTAAGAGAAGCAACATCTC (p.Leu747_Pro753delinsSer) 18%
EGFR	EXON 19 DELETION	c.2235_2249delGGAATTAAGAGAAGC (p.Glu746_Ala750del) 40%
EGFR	EXON 19 DELETION	c.2237_2255delAATTAAGAGAAGCAACATCinsT (p.Glu746_Ser752delinsVal) 27%
EGFR	EXON 19 DELETION	c.2237_2255delAATTAAGAGAAGCAACATCinsT (p.Glu746_Ser752delinsVal) 18%
EGFR	EXON 19 DELETION	c.2240_2257delTAAGAGAAGCAACATCTC (p.Leu747_Pro753delinsSer) 17%
EGFR	EXON 19 DELETION	c.2235_2249delGGAATTAAGAGAAGC (p.Glu746_Ala750del) 56%
EGFR	EXON 19 DELETION	c.2239_2264delinsGCAACATCTCCGGAAGG (p.Leu747_Ala755delinsAlaThrSerProGluGly) 58%
EGFR	EXON 19 DELETION	c.2235_2249delGGAATTAAGAGAAGC (p.Glu746_Ala750del) 22%
EGFR	EXON 19 DELETION	c.2239_2248delTTAAGAGAAGinsC (p.Leu747_Ala750delinsPro) 68%
EGFR	EXON 19 DELETION	c.2235_2249delGGAATTAAGAGAAGC (p.Glu746_Ala750del) 25%
EGFR	EXON 19 DELETION	c.2239_2248delTTAAGAGAAGinsC (p.Leu747_Ala750delinsPro) 11%
EGFR	EXON 19 DELETION	c.2236_2250delGAATTAAGAGAAGCA (p.Glu746_Ala750del) 36%
EGFR	EXON 19 DELETION	c.2235_2249delGGAATTAAGAGAAGC (p.Glu746_Ala750del) 2%
EGFR	EXON 19 DELETION	c.2235_2249delGGAATTAAGAGAAGC (p.Glu746_Ala750del) 56%
EGFR	EXON 19 DELETION	c.2235_2249delGGAATTAAGAGAAGC (p.Glu746_Ala750del) 31%
EGFR	EXON 19 DELETION	c.2236_2250delGAATTAAGAGAAGCA (p.Glu746_Ala750del) 32%
EGFR	EXON 19 DELETION	c.2235_2249delGGAATTAAGAGAAGC (p.Glu746_Ala750del) 10%
EGFR	EXON 19 DELETION	c.2235_2249delGGAATTAAGAGAAGC (p.Glu746_Ala750del) 41%
EGFR	EXON 19 DELETION	c.2237_2255delAATTAAGAGAAGCAACATCinsT (p.Glu746_Ser752delinsVal)
EGFR	EXON 19 DELETION	c.2237_2255delAATTAAGAGAAGCAACATCinsT (p.Glu746_Ser752delinsVal)
EGFR	EXON 19 DELETION	c.2235_2249delGGAATTAAGAGAAGC (p.Glu746_Ala750del) 44%
EGFR	EXON 19 DELETION	c.2236_2250delGAATTAAGAGAAGCA (p.Clu746_Ala750del) 61%
EGFR	EXON 19 OTHER	c.2281G>A (p.Asp761Asn)
EGFR	EXON 19 OTHER	c.2239_2260delinsCAAC (p.Leu747_Lys754delinsGlnGln) 19%
EGFR	EXON 19 OTHER	c.2240T>C (p.Leu747Ser)
EGFR	EXON 20 INSERTION	c.2314_2319dupCCCCAC (p.Pro772_His773dup) 32%
EGFR	EXON 20 INSERTION	c.2308_2309insGGTTCGTGG (p.Val769_Asp770insGlyPheVal) 59%
EGFR	EXON 20 INSERTION	c.2311delinsCACCCCC (p.Asn771delinsHisProHis) 26%
EGFR	EXON 20 INSERTION	c.2312_2313insTGT (p.Asn771_Pro772insVal) (19%)
EGFR	EXON 20 INSERTION	(p.Ala763_Tyr764insPheGlnGluAla) 45%
EGFR	EXON 20 INSERTION	c.2311_2312insGGG (p.Asn771delinsArgAsp) (10%)
EGFR	EXON 20 INSERTION	c.2300_2308dupCCAGCGTGG (p.Ala767_Val769dup) 13%
EGFR	EXON 20 INSERTION	c.2319_2320insACCCAC (p.His773_Val774insThrHis) 34%
EGFR	EXON 20 INSERTION	c.2319_2320insACCCAC (p.His773_Val774insThrHis) 11%
EGFR	EXON 20 INSERTION	c.2311_2313delinsCACGTG (p.ASN771delinsHisVal)49%
EGFR	EXON 20 INSERTION	c.2303_2311dupGCGTGGACA (p.Ser768_Asp770dup) 42%
EGFR	EXON 20 INSERTION	c.2303_2311dupGCGTGGACA (p. ser768_Asp770dup) 33%
EGFR	EXON 20 INSERTION	c.2319_2320insTAC (p.His773_Val774insTyr) 18%
EGFR	EXON 20 INSERTION	c.2284-5_2290dup (p.Ala763_Tyr764insPheGlnGluAla) 26%
EGFR	EXON 20 INSERTION	c.2307_2315dupGGACAACCC (p.Asp770_Pro772dup) 23%
EGFR	EXON 20 INSERTION	c.2316delinsTCACGTG (p.His773_Val774dup) 1%
EGFR	EXON 20 INSERTION	c.2300_2308dupCCAGCGTGG (p.Ala767_Val769dup) 32%
EGFR	EXON 20 INSERTION	c.2310_2311insGGG (p.Asp770_Asn771insGly) 30%
EGFR	EXON 20 INSERTION	c.2308_2309insGCGAGG (p.Val769_Asp770insGlyGlu) 29%

EGFR	EXON 20 OTHER	c.2300_2308dupCCAGCGTGG (p.Ala767_Val769dup) 28%
EGFR	EXON 20 OTHER	c.2303_2311dupGCGTGGACA (p.Ser768_Asp770dup) 51%
EGFR	EXON 20 OTHER	c.2389T>A (p.Cys797Gly) 6%
EGFR	EXON 20 OTHER	(p.Gly779Phe) 13%
EGFR	EXON 20 OTHER	c.2432C>T (p.Ser811Phe) 54%
EGFR	EXON 20 OTHER	c.2335_2336delGGinsTT (p.Gly779Phe) 26%
EGFR	EXON 20 OTHER	c.2300_2308dupCCAGCGTGG (p.Ala767_Val769dup) 8%
EGFR	EXON 20 OTHER	c.2303_2311dupGCGTGGACA (p.Ser768_Asp770dup) 22%
EGFR	EXON 20 OTHER	c.2303_2311dupGCGTGGACA (p.Ser768_Asp770dup) 15%
EGFR	EXON 20 OTHER	c.2300_2308dupCCAGCGTGG (p.Ala767_Val769dup) 13%
EGFR	EXON 20 OTHER	c.2300_2308dupCCAGCGTGG (p.Ala767_Val769dup) 16%
EGFR	EXON 20 OTHER	c.2303G>T (p.Ser768Ile) 33%
EGFR	EXON 20 OTHER	c.2300C>T (p.Ala767Val)
EGFR	EXON 20 OTHER	c.2317_2322dupCACGTG (p.His773_Val774dup) 29%
EGFR	EXON 20 OTHER	c.2303G>T (p.Ser768Ile) 9%
EGFR	EXON 20 OTHER	c.2303G>T (p.Ser768Ile) 9%
EGFR	EXON 20 OTHER	c.2303_2311dupGCGTGGACA (p.Ser768_asp770dup) 37%
EGFR	EXON 21 OTHER	c.2612C>G (p.Ala871Gly)
EGFR	EXON 21 OTHER	c.2582T>A (p.Leu861Gln) 8%
EGFR	EXON 21 OTHER	c.2582T>A (p.Leu861Gln) 36%
EGFR	EXON 21 OTHER	c.2543C>T (p.Pro848Leu) 50%
EGFR	EXON 21 OTHER	c.2579A>T (p.Lys860Ile) 38%
EGFR	EXON 21 OTHER	c.2609A>G (p.His870Arg) 53%
EGFR	L858R	c.2573T>G (p.Leu858Arg)
EGFR	L858R	c.2573T>G (p.Leu858Arg) 7%
EGFR	L858R	c.2573T>G (p.Leu858Arg)
EGFR	L858R	c.2573T>G (p. Leu858Arg) 50%
EGFR	L858R	c.2573GT>G (p.Leu858Arg) 31%
EGFR	L858R	c.2573T>G (p. Leu858Arg) 27%
EGFR	L858R	c.2573T>G (p.Leu858Arg)
EGFR	L858R	c.2573T>G (p.Leu858Arg) 43%
EGFR	L858R	c.2573T>G (p.Leu858Arg) 10%
EGFR	L858R	c.2573T>G (p.Leu858Arg) 73%
EGFR	L858R	c.2573T>G (p.Leu858Arg)
EGFR	L858R	c.2573T>G (p.Leu858Arg)
EGFR	L858R	c.2573T>G (p. Leu858Arg) 31%
EGFR	L858R	c.2573T>G (p.Leu858Arg)
EGFR	L858R	c.2573T>G (p.Leu858Arg) 13%
EGFR	L858R	c.2573T>G (p.Leu858Arg)
EGFR	L858R	c.2573T>G (p.Leu858Arg) 29%
EGFR	L858R	c.2573T>G (p.Leu858Arg) 18%
EGFR	L858R	c.2573T>G (p.Leu858Arg) (37%)
EGFR	L858R	c.2573T>G (p.Leu858Arg) 30%
EGFR	L858R	c.2573T>G (p.Leu858Arg) 34%
EGFR	L858R	c.2573T>G (p.Leu858Arg) 15%
EGFR	L858R	c.2573T>G (p.Leu858Arg) 62%
EGFR	L858R	c.2573T>G (p.Leu858Arg) 19%

EGFR	L858R	c.2573GT>G (p.Leu858Arg) 11%
EGFR	L858R	c.2573T>G (p.Leu858Arg) 10%
EGFR	L858R	c.2573T>G (p.Leu858Arg) 21%
EGFR	L858R	c.2573T>G (p.Leu858Met) 8%
EGFR	L858R	c.2573T>G (p.Leu858Arg)
EGFR	L858R	c.2573T>G (p.Leu858Arg)
EGFR	L858R	c.2573T>G (p.Leu858Arg) 40%
EGFR	L858R	c.2573T>G (p.Leu858Arg) 22%
EGFR	L858R	c.2573T>G (p.Leu858Arg) 8%
EGFR	L858R	c.2573_2574delinsGT (p.Leu858Arg) 22%
EGFR	L858R	c.2573T>G (p.Leu858Arg) 54%
EGFR	L858R	c.2573T>G (p.Leu858Arg) 36%
EGFR	L858R	c.2573T>G (p.Leu858Arg) 34%
EGFR	L858R	c.2573T>G (p.Leu858Arg) 26%
EGFR	L858R	c.2573T>G (p.Leu858Arg)
EGFR	L858R	c.2573T>G (p.Leu858Arg) 30%
EGFR	L858R	c.2563T>G (p.Leu858Arg) 10%
EGFR	L858R	c.2573T>G (p.Leu858Arg) 20%
EGFR	L858R	c.2573T>G (p.Leu858Arg) 14%
EGFR	L858R	c.2573T>G (p.Leu858Arg) 12%
EGFR	L858R	c.2573T>G (p.Leu858Arg) 41%
EGFR	L858R	c.2573T>G (p.Leu858Arg) 18%
EGFR	L858R	c.2573T>G (p.Leu858Arg) 31%
EGFR	L858R	c.2573T>G (p.Leu858Arg) 24%
EGFR	L858R	c.2573T>G (p.Leu858Arg) 23%
EGFR	L858R	c.2573T>G (p.Leu858Arg) 12%
EGFR	L858R	c.2573T>G (p.Leu858Arg) 20%
EGFR	L858R	c.2573T>G (p.Leu858Arg) 14%
EGFR	L858R	c.2573T>G (p.Leu858Arg) 67%
EGFR	L858R	c.2573T>G (p.Leu858Arg) 93%
EGFR	L858R	c.2573_2574delTGinsGT (p.Leu858Arg) 20%
EGFR	L858R	c.2573T>G (p.Leu858Arg) 27%
EGFR	L858R	c.2573T>G (p.Leu858Arg) 71%
EGFR	L858R	c.2573T>G (p.Leu858Arg) 10%
EGFR	L858R	c.2573T>G (p.Leu858Arg) 47%
EGFR	L858R	c.2572_2573delCTinsAG (p.Leu858Arg) 22%
EGFR	L858R	c.2573T>G (p.Leu858Arg) 31%
EGFR	L858R	c.2573T>G (p.Leu858Arg) 7%
EGFR	L858R	c.2573T>G (p.Leu858Arg) 10%
EGFR	L858R	c.2573T>G (p.Leu858Arg) 35%
EGFR	L858R	c.2573T>G (p.Leu858Arg) 9%
EGFR	L858R	c.2573T>G (p.Leu858Arg) 66%
EGFR	L858R	c.2573T>G (p.Leu858Arg) 27%
EGFR	L858R	c.2573T>G (p.Leu858Arg)
EGFR	L858R	c.2573T>G (p.Leu858Arg) 73%
EGFR	L858R	c.2573T>G (p.Leu858Arg) 59%
EGFR	L858R	c.2573T>G (p.Leu858Arg) (30%)

EGFR	L858R	c.2573T>G (p.Leu858Arg) 70%
EGFR	L858R	c.2573T>G (p.Leu858Arg) 18%
EGFR	L858R	c.2573T>G (p.Leu858Arg) 81%
EGFR	L858R	c.2573T>G (p.Leu858Arg) 91%
EGFR	L858R	c.2573T>G (p.Leu858Arg)
EGFR	L858R	c.2573T>G (p.Leu858Arg) 31%
EGFR	L858R	c.2573T>G (p.Leu858Arg) 24%
EGFR	L858R	c.2573T>G (p.Leu858Arg) 14%
EGFR	L858R	c.2573T>G (p.Leu858Arg) 30%
EGFR	L858R	c.2573T>G (p.Leu858Arg) 51%
EGFR	L858R	c.2573T>G (p.Leu858Arg) 23%
EGFR	L858R	c.2573T>G (p.Leu858Arg) 29%
EGFR	L858R	c.2573T>G (p.Leu858Arg) 22%
EGFR	L858R	c.2573T>G (p. Leu858Arg) 53%
EGFR	L858R	c.2573T>G (p.Leu858Arg) 28%
EGFR	L858R	c.2573T>G (p.Leu858Arg) 32%
EGFR	L858R	c.2573T>G (p.Leu858Arg) 16%
EGFR	L858R	c.2573T>G (p.Leu858Arg) 34%
EGFR	L858R	c.2573T>G (p.Leu858Arg) 18%
EGFR	L858R	c.2573T>G (p.Leu858Arg)
EGFR	L858R	c.2573T>G (p.Leu858Arg)
EGFR	L858R	c.2573T>G (p.Leu858Arg) 14%
EGFR	L858R	c.2573T>G (p.Leu858Arg) 21%
EGFR	L858R	c.2573T>G (p.Leu858Arg) 86%
EGFR	L858R	c.2573T>G (p.Leu858Arg) 11%
EGFR	L858R	c.2573T>G (p.Leu858Arg)
EGFR	L858R	c.2573T>G (p.Leu858Arg) 14%
EGFR	L858R	c.2573T>G (p.Leu858Arg) 59%
EGFR	L858R	c.2573T>G (p.Leu858Arg) 18%
EGFR	L858R	c.2573T>G (p.Leu858Arg) 25%
EGFR	L858R	c.2573T>G (p.Leu858Arg) 4%
EGFR	L858R	c.2573T>G (p.Leu858Arg) 27%
EGFR	L858R	c.2573T>G (p.Leu858Arg) 29%
EGFR	L858R	c.2573T>G (p.Leu858Arg) 59%
EGFR	L858R	c.2573T>G (p.Leu858Arg) 4%
EGFR	L858R	c.2573T>G (p.Leu858Arg) 20%
EGFR	L858R	c.2573T>G (p.Leu858Arg) 91%
EGFR	L858R	c.2573T>G (p.Leu858Arg) 43%
EGFR	L858R	c.2573T>G (p.Leu858Arg) 15%
EGFR	L858R	c.2573T>G (p.Leu858Arg) 21%
EGFR	L858R	c.2573T>G (p.Leu858Arg) 38%
EGFR	L858R	c.2573T>G (p.Leu858Arg) 34%
EGFR	L858R	c.2573T>G (p.Leu858Arg) 38%
EGFR	L858R	c.2573T>G (p.Leu858Arg) 36%
EGFR	L858R	c.2573T>G (p.Leu858Arg) 35%
EGFR	L858R	c.2573T>G (p.Leu858Arg) 36%
EGFR	L858R	c.2573T>G (p.Leu858Arg) 83%

EGFR	L858R	c.2573T>G (p.Leu858Arg) 36%
EGFR	L858R	c.2573T>G (p.Leu858Arg) 27%
EGFR	L858R	c.2573T>G (p.Leu858Arg) 43%
EGFR	L858R	c.2573T>G (p.Leu858Arg) 55%
EGFR	L858R	c.2573T>G (p.Leu858Arg) 94%
EGFR	L858R	c.2573T>G (p.Leu858Arg) 12%
EGFR	L858R	c.2573T>G (p.Leu858Arg) 13%
EGFR	L858R	c.2573T>G (p.Leu858Arg) 45%
EGFR	L858R	c.2573T>G (p.Leu858Arg) 54%
EGFR	L858R	c.2573T>G (p.Leu858Arg) 7%
EGFR	L858R	c.2573T>G (p.Leu858Arg) (27%)
EGFR	L858R	c.2573T>G (p.Leu858Arg) 26%
EGFR	L858R	c.2573T>G (p.Leu858Arg) 26%
EGFR	L858R	c.2573T>G (p.Leu858Arg) 24%
EGFR	L858R	c.2573T>G (p.Leu858Arg) 35%
EGFR	L858R	c.2573T>G (p.Leu858Arg) 15%
EGFR	L858R	c.2573T>G (p.Leu858Arg) 24%
EGFR	L858R	c.2573T>G (p.Leu858Arg) 32%
EGFR	L858R	c.2573T>G (p.Leu858Arg) 31%
EGFR	L858R	c.2573T>G (p.Leu858Arg) 53%
EGFR	L858R	c.2573T>G (p.Leu858Arg) 47%
EGFR	L858R	c.2573T>G (p.Leu858Arg) 64%
EGFR	L858R	c.2573T>G (p.Leu858Arg) 13%
EGFR	L858R	c.2572_2573delCTinsAG (p.Leu858Arg) 30%
EGFR	L858R	c.2573_2574delTGinsGT (p.Leu858Arg) 20%
EGFR	L858R	c.2573T>G (p.Leu858Arg) 13%
EGFR	L861Q	c.2582T>A (p.Leu861Gln) 18%
EGFR	L861Q	c.2582T>A (p.Leu861Gln)
EGFR	L861Q	c.2582T>A (p.Leu861Gln) 15%
EGFR	L861Q	c.2582T>A (p.Leu861Gln) 83%
EGFR	L861Q	c.2582T>A (p.Leu861Gln) 9%
EGFR	L861Q	c.2582T>A (p.Leu861GLN) 10%
EGFR	L861Q	c.2582T>A (p.Leu861Gln) 25%
EGFR	L861Q	c.2582T>A (p.Leu861Gln) 22%
EGFR	L861Q	c.2582T>A (p.Leu861Gln) (73%)
EGFR	L861Q	c.2582T>A (p.Leu861Gln) 40%
EGFR	OTHER	c.1451C>T (p. Ser484Phe) 50%
EGFR	T790M	c.2369C>T (p.Thr790Met) 50%
EGFR	T790M	c.2369C>T (p.Thr790Met) 26%
EGFR	T790M	c.2369C>T (p.Thr790Met) 1%
EGFR	T790M	c. 2369C>T (p.Thr790Met) 27%
EGFR	T790M	c. 2369C>T (p.Thr790Met) 9%
EGFR	T790M	c.2369C>T (p.Thr790Met) 15%
EGFR	T790M	c.2369C>T (p.Thr790Met) 31%
EGFR	T790M	c.2369C>T (p.Thr790Met) 25%
EGFR	T790M	c.2369C>T (p.Thr790Met) 34%
EGFR	T790M	c.2369C>T (p.Thr790Met) 6%

EGFR	T790M	c.2369C>T (p.Thr790Met) 29%
EGFR	T790M	c.2369C>T (p.Thr790Met) 63%
EGFR	T790M	c.2369C>T (p.Trh790Met) 12%
EGFR	T790M	c.2369C>T (p.Thr790Met) 14%
EGFR	T790M	c.2369C>T (p.Thr790Met) 6%
EGFR	T790M	c.2369C>T (p.Thr790Met) 2%
ERBB2	EXON 20	c.2313_2324dupATACGTGATGGC (p. Tyr772_Ala775dup) 16%
ERBB2	EXON 20	c.2313_2324dupATACGTGATGGC (p.Tyr772_Ala775dup) 34%
ERBB2	EXON 20	c.2326_2327insTGT (p.Gly776delinsValCys) 15%
ERBB2	EXON 20	Unknown
ERBB2	EXON 20	c.2313_2324dupATACGTGATGGC (p.Tyr772_Ala775dup) 97%
ERBB2	EXON 20	c.2314_2325dupTACGTGATGGCT (p.Tyr772_Ala775dup) 26%
ERBB2	EXON 20	Unknown
ERBB2	EXON 20	c.2313_2324dupATACGTGATGGC (p.Tyr772_Ala775dup) 15%
ERBB2	EXON 20	c.2313_2324dupATACGTGATGGC (p.Tyr772_Ala775dup) 13%
ERBB2	EXON 20	c.2313_2324dupATACGTGATGGC (p.Tyr772_Ala775dup) 41%
ERBB2	EXON 20	Unknown
ERBB2	EXON 20	c.2313_2324dupATACGTGATGGC (p.Tyr772_Ala775dup) 63%
ERBB2	EXON 20	c.2313_2324dupATACGTGATGGC (p.Tyr772_Ala775dup)
ERBB2	EXON 20	Unknown
ERBB2	EXON 20	Unknown
ERBB2	EXON 20	c.2313_2324dupATACGTGATGGC (p.Tyr772_Ala775dup) 48%
ERBB2	EXON 20	c.2313_2324dupATACGTGATGGC (p.Tyr772_Ala775dup)
ERBB2	EXON 20	c.2313_2324dupATACGTGATGGC (p.Tyr772_Ala775dup) 63%
ERBB2	EXON 20	c.2313_2324dupATACGTGATGGC (p.Tyr772_Ala775dup) 55%
ERBB2	EXON 20	c.2313_2324dupATACGTGATGGC (p.Tyr772_Ala775dup) 28%
ERBB2	EXON 20	c.2313_2324dup(ATACGTGATGGC (p.Tyr772_Ala775dup) 40%
ERBB2	EXON 20	c.2326_2331delinsAGTGTGTGT (p.Gly776_Val777delinsServValCys) 32%
ERBB2	EXON 20	c.2331_2339dupGGGCTCCCC (p.Gly778_Pro780dup) 32%
ERBB2	OTHER	c.2521C>A (p.Leu841Ile) 9%
ERBB2	OTHER	c.2264T>C (p.Leu755Ser) 23%
ERBB2	OTHER	c.2263_2264delTTinsGC (p.Leu755Ala)
ERBB2	OTHER	c.2263_2265delTTGinsCCC (p.Leu755Pro) 46%
ERBB2	TMD	c.1976T>A (p.Val659Asp)
ERBB2	TMD	c.1976_1977delTTinsAA (p.Val659Glu) 32%
ERBB2	TMD	c.1979G>A (p.Gly660Asp)
ERBB2	TMD	c.1976_1977delTTinsAA (p.Val659Glu)
KRAS	G12A	c.35G>C (p.Gly12Ala)
KRAS	G12A	c.35G>C (p.Gly12Ala) 37%
KRAS	G12A	c.35G>C (p.Gly12Ala) 26%
KRAS	G12A	c.35G>C (p.Gly12Ala) 8%
KRAS	G12A	c.35G>C (p.Gly12Ala) 24%
KRAS	G12A	c.35G>C (p.Gly12Ala) 17%
KRAS	G12A	c.35G>C (p.Gly12Ala) 7%
KRAS	G12A	c.35G>C (p.Gly12Ala) 24%
KRAS	G12A	c.35G>C (p.Gly12Ala) 22%
KRAS	G12A	c.35G>C (p.Gly12Ala) 32%

KRAS	G12A	c.35G>C (p.Gly12Ala) 13%
KRAS	G12A	c.35G>C (p.Gly12Ala) 9%
KRAS	G12A	c.35G>C (p.Gly12Ala) 15%
KRAS	G12A	c.35G>C (p.Gly12Ala) 17%
KRAS	G12A	c.35G>C (p.Gly12Ala) 37%
KRAS	G12A	c.35G>C (p.Gly12Ala)
KRAS	G12A	c.35G>C (p.Gly12Ala)
KRAS	G12A	c.35G>C (p.Gly12Ala) 16%
KRAS	G12A	c.35G>C (p.Gly12Ala) 10%
KRAS	G12A	c.35G>C (p.Gly12Ala) 50%
KRAS	G12A	c.35G>C (p.Gly12Ala) 12%
KRAS	G12A	c.35G>C (p.Gly12Ala) 71%
KRAS	G12A	c.35G>C (p.Gly12Ala) 29%
KRAS	G12A	c.35G>C (p.Gly12Ala) 10%
KRAS	G12A	c.35G>C (p.Gly12Ala) 22%
KRAS	G12A	c.35G>C (p. Gly12Ala) 5%
KRAS	G12A	c.35G>C (p.Gly12Ala) 7%
KRAS	G12A	c.35G>C (p.Gly12Ala) 3%
KRAS	G12A	c.35G>C (p.Gly12Ala) 7%
KRAS	G12A	c.35G>C (p.Gly12Ala) 31%
KRAS	G12A	c.35G>C (p.Gly12Ala) 23%
KRAS	G12A	c.35G>C (p.Gly12Ala) 16%
KRAS	G12A	c.35G>C (p.Gly12Ala) 71%
KRAS	G12A	c.35G>C (p.Gly12Ala)
KRAS	G12A	c.35G>C (p.Gly12Ala)
KRAS	G12A	c.35G>C (p.Gly12Ala) 29%
KRAS	G12A	c.35G>C (p.Gly12Ala) 63%
KRAS	G12A	c.35G>C (p.Gly12Ala) 6%
KRAS	G12A	c.35G>C (p.Gly12Ala) 41%
KRAS	G12A	c.35 G>C (p.Gly12Ala) 8%
KRAS	G12A	c.35G>C (p.Gly12Ala) 12%
KRAS	G12A	c.35G>C (p.Gly12Ala) 39%
KRAS	G12C	c.34G>T (p.Gly12Cys) 24%
KRAS	G12C	c.34G>T (p.Gly12Cys) 14%
KRAS	G12C	c.34G>T (p.Gly12Cys) 21%
KRAS	G12C	c.34G>T (p.Gly12Cys) 24%
KRAS	G12C	c.34G>T (p.Gly12Cys) 20%
KRAS	G12C	c.34G>T (p.Gly12Cys) 12%
KRAS	G12C	c.34G>T (p.Gly12Cys)
KRAS	G12C	c.34G>T (p.Gly12Cys) 40%
KRAS	G12C	c.34G>T (p.Gly12Cys) 37%
KRAS	G12C	c.34G>T (p.Gly12Cys) 15%
KRAS	G12C	c.34G>T (p.Gly12Cys)
KRAS	G12C	c.34G>T (p.Gly12Cys)
KRAS	G12C	c.34G>T (p.Gly12Cys) 21%
KRAS	G12C	c.34G>T (p.Gly12Cys) 20%
KRAS	G12C	c.34G>T (p.Gly12Cys) 7%

KRAS	G12C	c.34G>T (p.Gly12Cys)
KRAS	G12C	c.34G>T (p.Gly12Cys)
KRAS	G12C	c.34G>T (p.Gly12Cys)
KRAS	G12C	c.34G>T (p.Gly12Cys) 9%
KRAS	G12C	c.34G>T (p.Gly12Cys) 8%
KRAS	G12C	c.34G>T (p.Gly12Cys) 31%
KRAS	G12C	c.34G>T (p.Gly12Cys) 23%
KRAS	G12C	c.34G>T (p.Gly12Cys)
KRAS	G12C	c.34G>T (p.Gly12Cys) 26%
KRAS	G12C	c.34G>T (p.Gly12Cys) 16%
KRAS	G12C	c.34G>T (p.Gly12Cys) 33%
KRAS	G12C	c.34G>T (p.Gly12Cys) 11%
KRAS	G12C	c.34G>T (p.Gly12Cys) 26%
KRAS	G12C	c.34G>T (p.Gly12Cys) 33%
KRAS	G12C	c.34G>T (p.Gly12Cys) 16%
KRAS	G12C	c.34G>T (p. Gly12Cys) 72%
KRAS	G12C	c.34G>T (p.Gly12Cys) 53%
KRAS	G12C	c.34G>T (p.Gly12Cys) 73%
KRAS	G12C	c.34G>T (p.Gly12Cys) 78%
KRAS	G12C	c.34G>T (p.Gly12Cys) 11%
KRAS	G12C	c.34G>T (p.Gly12Cys) 32%
KRAS	G12C	c.34G>T (p.Gly12Cys) 11%
KRAS	G12C	c.34G>T (p.Gly12Cys)
KRAS	G12C	c.34G>T (p.Gly12Cys) 50%
KRAS	G12C	c.34G>T (p.Gly12Cys)
KRAS	G12C	c.34G>T (p.Gly12Cys) 17%
KRAS	G12C	c.34G>T (p.Gly12Cys) 30%
KRAS	G12C	c.34G>T (p. Gly12Cys) 27%
KRAS	G12C	c.34G>T (p.Gly12Cys) 61%
KRAS	G12C	c.34G>T (p.Gly12Cys)
KRAS	G12C	c.34G>T (p.Gly12Cys) 12%
KRAS	G12C	c.34G>T (p.Gly12Cys) 10%
KRAS	G12C	c.34G>T (p.Gly12Cys) 87%
KRAS	G12C	c.34G>T (p.Gly12Cys) 14%
KRAS	G12C	c.34G>T (p.Gly12Cys) 19%
KRAS	G12C	c.34G>T (p.Gly12Cys) 18%
KRAS	G12C	c.34G>T (p.Gly12Cys) 30%
KRAS	G12C	c.34G>T (p.Gly12Cys) 5%
KRAS	G12C	c.34G>T (p.Gly12Cys) 17%
KRAS	G12C	c.34G>T (p.Gly12Cys) 17%
KRAS	G12C	c.34G>T (p.Gly12Cys) 35%
KRAS	G12C	c.34G>T (p.Gly12Cys) 11%
KRAS	G12C	c.34G>T (p.Gly12Cys) 14%
KRAS	G12C	c.36G>T (p.Gly12Cys) 76%
KRAS	G12C	c.34G>T (p. Gly12Cys) 18%
KRAS	G12C	c.34G>T (p.Gly12Cys) 22%
KRAS	G12C	c.34G>T (p.Gly12Cys) 8%

KRAS	G12C	c.34G>T (p.Gly12Cys) 54%
KRAS	G12C	c.34G>T (p.Gly12Cys) 4%
KRAS	G12C	c.34G>T (p.Gly12Cys) 24%
KRAS	G12C	c.34G>T (p.Gly12Cys) 13%
KRAS	G12C	c.34G>T (p.Gly12Cys) 38%
KRAS	G12C	c.34G>T (p.Gly12Cys) 37%
KRAS	G12C	c.34G>T (p. Gly12Cys) 14%
KRAS	G12C	c.34G>T (p.Gly12Cys) 25%
KRAS	G12C	c.34G>T (p.Gly12Cys) 20%
KRAS	G12C	c.34G>T (p.Gly12Cys) 18%
KRAS	G12C	c.34G>T (p.Gly12Cys) 5%
KRAS	G12C	c.34G>T (p.Gly12Cys) 27%
KRAS	G12C	c.34G>T (p.Gly12Cys) 28%
KRAS	G12C	c.34G>T (p.Gly12Cys) 17%
KRAS	G12C	c.34G>T (p.Gly12Cys) 5%
KRAS	G12C	c.34G>T (p.Gly12Cys) 67%
KRAS	G12C	c.34G>T (p.Gly12Cys) 17%
KRAS	G12C	c.34G>T (p. Gly12Cys) 9%
KRAS	G12C	c34G>T (p.Gly12Cys) 31%
KRAS	G12C	c.34G>T (p. Gly12Cys) 15%
KRAS	G12C	c.34G>T (p.Gly12Cys) 43%
KRAS	G12C	c.34G>T (p.Gly12Cys) 15%
KRAS	G12C	c.34G>T (p.Gly12Cys) 55%
KRAS	G12C	c.34G>T (p.Gly12Cys) 25%
KRAS	G12C	c.34G>T (p. Gly12Cys) 18%
KRAS	G12C	c.34G>T (p.Gly12Cys)
KRAS	G12C	c.34G>T (p. Gly12Cys) 23%
KRAS	G12C	c.34G>T (p.Gly12Cys) 16%
KRAS	G12C	c.34G>T (p. Gly12Cys) 20%
KRAS	G12C	c.34G>T (pGly12Cys) 10%
KRAS	G12C	c.34G>T (p.Gly12Cys)
KRAS	G12C	c.34G>T (p.Gly12Cys) 39%
KRAS	G12C	c.34G>T (p.Gly12Cys) 6%
KRAS	G12C	c.34G>T (p.Gly12Cys) 8%
KRAS	G12C	c.34G>T (p.Gly12Cys)
KRAS	G12C	c.34G>T (p. Gly12Cys) 57%
KRAS	G12C	c.34G>T (p.Gly12Cys) 26%
KRAS	G12C	c.34G>T (p.Gly12Cys) 51%
KRAS	G12C	c.34G>T (p.Gly12Cys) 30%
KRAS	G12C	c.34G>T (p.Gly12Cys) 36%
KRAS	G12C	c.34G>T (p.Gly12Cys) 59%
KRAS	G12C	c.34G>T (p.Gly12Cys) 19%
KRAS	G12C	c.34G>T (p.Gly12Cys) 39%
KRAS	G12C	c.34G>T (p.Gly12Cys) 70%
KRAS	G12C	c.34G>T (p.Gly12Cys) 29%
KRAS	G12C	c.34G>T (p.Gly12Cys) 24%
KRAS	G12C	c.34G>T (p.Gly12Cys) 91%

KRAS	G12C	c.34G>T (p.Gly12Cys) 22%
KRAS	G12C	c.34G>T (p.Gly12Cys) 19%
KRAS	G12C	c.34G>T (p.Gly12Cys) 21%
KRAS	G12C	c.34G>T (p.Gly12Cys) 21%
KRAS	G12C	c.34G>T (p.Gly12Cys) 34%
KRAS	G12C	c.34G>T (p.Gly12Cys) 75%
KRAS	G12C	c.34G>T (p.Gly12Cys) 59%
KRAS	G12C	c.34G>T (p.Gly12Cys) 61%
KRAS	G12C	c.34G>T (p.Gly12Cys) 7%
KRAS	G12C	c.34G>T (p.Gly12Cys) 56%
KRAS	G12C	c.34G>T (p.Gly12Cys)
KRAS	G12C	c.34G>T (p.Gly12Cys) 45%
KRAS	G12C	c.34G>T (p.Gly12Cys) 27%
KRAS	G12C	c.34G>T (p.Gly12Cys) 27%
KRAS	G12C	c.34G>T (p.Gly12Cys) 30%
KRAS	G12C	c.34G>T (p.Gly12Cys) 41%
KRAS	G12C	c.34G>T (p.Gly12Cys)
KRAS	G12C	c.34G>T (p.Gly12Cys)
KRAS	G12C	c.34G>T (p.Gly12Cys) 21%
KRAS	G12C	c.34G>T (p.Gly12Cys) 55%
KRAS	G12C	c.34G>T (p.Gly12Cys)
KRAS	G12C	c.34G>T (p.Gly12Cys)
KRAS	G12C	c.34G>T (p.Gly12Cys) 28%
KRAS	G12C	c.34G>T (p. Gly12Cys) 66%
KRAS	G12C	c.34G>T (p.Gly12Cys) 28%
KRAS	G12C	c.34G>T (p.Gly12Cys)
KRAS	G12C	c.34G>T (p.Gly12Cys) 61%
KRAS	G12C	c.34G>T (p.Gly12Cys) 9%
KRAS	G12C	c.34G>T (p.Gly12Cys) 9%
KRAS	G12C	c.34G>T (p.Gly12Cys) 16%
KRAS	G12C	c.34G>T (p.Gly12Cys) 17%
KRAS	G12C	c.34G>T (p.Gly12Cys) 45%
KRAS	G12C	c.34G>T (p.Gly12Cys) 18%
KRAS	G12C	c.34G>T (p.Gly12Cys) 27%
KRAS	G12C	c.34G>T (p.Gly12Cys) 13%
KRAS	G12C	c.34G>T (p.Gly12Cys) 25%
KRAS	G12C	c.34G>T (p.Gly12Cys) 52%
KRAS	G12C	c.34G>T (p.Gly12Cys) 25%
KRAS	G12C	c.34G>T (p.Gly12Cys) 29%
KRAS	G12C	c.34G>T (p.Gly12Cys) 28%
KRAS	G12C	c.34G>T (p.Gly12Cys) 42%
KRAS	G12C	c.34G>T (p.Gly12Cys) 44%
KRAS	G12C	c.34G>T (p.Gly12Cys) 14%
KRAS	G12C	c.34G>T (p.Gly12Cys) 46%
KRAS	G12C	c.34G>T (p.Gly12Cys) 22%
KRAS	G12C	c.34G>T (p.Gly12Cys) 57%
KRAS	G12C	c.34G>T (p.Gly12Cys) 33%

KRAS	G12C	c.34G>T (p.Gly12Cys) 19%
KRAS	G12C	c.34G>T (p.Gly12Cys) 10%
KRAS	G12C	c.34G>T (p.Gly12Cys) 5%
KRAS	G12C	c.34G>T (p.Gly12Cys) 13%
KRAS	G12C	c.34G>T (p.Gly12Cys) 52%
KRAS	G12C	c.34G>T (p.Gly12Cys) 11%
KRAS	G12C	c.34G>T (p.Gly12Cys) 34%
KRAS	G12C	c.34G>T (p. Gly12Cys) 31%
KRAS	G12C	c.34G>T (p.Gly12Cys) 12%
KRAS	G12D	c.35G>A (p.Gly12Asp)
KRAS	G12D	c.35G>A (p.Gly12Asp)
KRAS	G12D	c.35G>A (p.Gly12Asp)
KRAS	G12D	c.35G>A (p.Gly12Asp) 43%
KRAS	G12D	c.35G>A (p.Gly12Asp) 51%
KRAS	G12D	c.35G>A (p.Gly12Asp)
KRAS	G12D	c.35G>A (p.Gly12Asp) 26%
KRAS	G12D	c.35G>A (p.Gly12Asp)
KRAS	G12D	c.35G>A (p.Gly12Asp) 26%
KRAS	G12D	c.35G>A (p.Gly12Asp) 11%
KRAS	G12D	c.35G>A (p.Gly12Asp) 8%
KRAS	G12D	c.35G>A (p.Gly12Asp)
KRAS	G12D	c.35G>A (p.Gly12Asp) 43%
KRAS	G12D	c.35G>A (p.Gly12Asp) 60%
KRAS	G12D	c.35G>A (p.Gly12Asp) 36%
KRAS	G12D	c.35G>A (p.Gly12Asp) 21%
KRAS	G12D	c.35G>A (p.Gly12Asp) 30%
KRAS	G12D	c.35G>A (p.Gly12Asp) 25%
KRAS	G12D	c.35G>A (p.Gly12Asp) 27%
KRAS	G12D	c.35G>A (p.Gly12Asp) 68%
KRAS	G12D	c.35G>A (p.Gly12Asp) 18%
KRAS	G12D	c.35G>A (p.Gly12Asp) 23%
KRAS	G12D	c.35G>A (p.Gly12Asp) 18%
KRAS	G12D	c.35G>A (p.Gly12Asp)
KRAS	G12D	c.35G>A (p.Gly12Asp) 57%
KRAS	G12D	c.35G>A (p.Gly12Asp)
KRAS	G12D	c.35G>A (p. Gly12Asp) 21%
KRAS	G12D	c.35G>A (p.Gly12Asp) 64%
KRAS	G12D	c.35G>A (p.Gly12Asp) 41%
KRAS	G12D	c.35G>A (p.Gly12Asp) 23%
KRAS	G12D	c.35G>A (p.Gly12Asp) 12%
KRAS	G12D	c.35G>A (p.Gly12Asp) 10%
KRAS	G12D	c.35G>A (p.Gly12Asp) 29%
KRAS	G12D	c.35G>A (p.Gly12Asp)
KRAS	G12D	c.35G>A (p.Gly12Asp) 30%
KRAS	G12D	c.35G>A (p.Gly12Asp) 16%
KRAS	G12D	c.35G>A (p. Gly12Asp) 29%
KRAS	G12D	c.35G>A (p.Gly12Asp)

KRAS	G12D	c.35G>A (p.Gly12Asp) 24%
KRAS	G12D	c.35G>A (p.Gly12Asp) 72%
KRAS	G12D	c.35G>A (p.Gly12Asp) 13%
KRAS	G12D	c.35G>A (p.Gly12Asp)
KRAS	G12D	c.35G>A (p.Gly12Asp) 40%
KRAS	G12D	c.35G>A (p. Gly12Asp) 18%
KRAS	G12D	c.35G>A (p.Gly12Asp)
KRAS	G12D	c.35G>A (p.Gly12Asp) 12%
KRAS	G12D	c.35G>A (p.Gly12Asp) 3%
KRAS	G12D	c.35G>A (p.Gly12Asp) 13%
KRAS	G12D	c.35G>A (p.Gly12Asp)
KRAS	G12D	c.35G>A (p.Gly12Asp) 38%
KRAS	G12D	c.35G>A (p.Gly12Asp) 24%
KRAS	G12D	c.36G>A (p.Gly12Asp) 29%
KRAS	G12D	c.35G>A (p.Gly12Asp) 14%
KRAS	G12D	c.35G>A (p.Gly12Asp) 11%
KRAS	G12D	c.35G>A (p.Gly12Asp) 73%
KRAS	G12D	c.35G>A (p.Gly12Asp) 20%
KRAS	G12D	c.35G>A (p.Gly12Asp) 13%
KRAS	G12D	c.35G>A (p.Gly12Asp) 63%
KRAS	G12D	c.35G>A (p.Gly12Asp) 18%
KRAS	G12D	c.35G>A (p.Gly12Asp) 3%
KRAS	G12D	c.35G>A (p.Gly12Asp) 72%
KRAS	G12D	c.35G>A (p.Gly12Asp)
KRAS	G12D	c.35G>A (p.Gly12Asp) 23%
KRAS	G12D	c.35G>A (p.Gly12Asp)
KRAS	G12D	c.35G>A (p.Gly12Asp)
KRAS	G12D	c.35G>A (p.Gly12Asp) 8%
KRAS	G12D	c.35G>A (p.Gly12Asp)
KRAS	G12D	c.35G>A (p.Gly12Asp) 35%
KRAS	G12D	c.35G>A (p.Gly12Asp) 25%
KRAS	G12D	c.35G>A (p.Gly12Asp) 15%
KRAS	G12D	c.35G>A (p.Gly12Asp) 15%
KRAS	G12D	c.35G>A (p.Gly12Asp) 25%
KRAS	G12D	c.35G>A (p.Gly12Asp) 26%
KRAS	G12D	c.35G>A (p.Gly12Asp) 18%
KRAS	G12D	c.35G>A (p.Gly12Asp) 58%
KRAS	G12D	c.35G>A (p.Gly12Asp) 22%
KRAS	G12D	c.35G>A (p.Gly12Asp) 30%
KRAS	G12D	c.35G>A (p.Gly12Asp) 24%
KRAS	G12D	c.35G>A (p.Gly12Asp) 21%
KRAS	G12D	c.35G>A (p.Gly12Asp) 35%
KRAS	G12D	c.35G>A (p.Gly12Asp) 8%
KRAS	G12D	c.35G>A (p.Gly12Asp)
KRAS	G12D	c.35G>A (p.Gly12Asp) 49%
KRAS	G12D	c.35G>A (p.Gly12Asp)
KRAS	G12F	c.34_35delGGinsTT (p.Gly12Phe) 12%

KRAS	G12F	c.34_35delGGinsTT (p.Gly12Phe) 39%
KRAS	G12F	c.34_35delGGinsTT (p.Gly12Phe) 9%
KRAS	G12F	c.34_35delinsTT (p. Gly12Phe) 41%
KRAS	G12F	c.34_35delGGinsTT (p.Gly12Phe) 9%
KRAS	G12F	c.34_35delGGinsTT (p.Gly12Phe) 42%
KRAS	G12F	c.34_35delGGinsTT (p.Gly12Phe) 13%
KRAS	G12F	c.34_35delGGinsTT (p.Gly12Phe) 51%
KRAS	G12F	c.34_35delGGinsTT (p.Gly12Phe) 29%
KRAS	G12R	c.34G>C (p.Gly12Arg) 6%
KRAS	G12R	c.34G>C (p.Gly12Arg) 21%
KRAS	G12R	c.34G>C (p.Gly12Arg) 31%
KRAS	G12R	c.34G>C (p. Gly12Arg) 15%
KRAS	G12R	c.34G>C (p.Gly12Arg) 5%
KRAS	G12V	c.35G>T (p.Gly12Val) 44%
KRAS	G12V	c.35G>T (p.Gly12Val) 35%
KRAS	G12V	c.33_35delTGGinsGGT (p. Gly12Val) 15%
KRAS	G12V	c.35G>T (p.Gly12Val) 4%
KRAS	G12V	c.35G>T (p.Gly12Val) 6%
KRAS	G12V	c.35G>T (p.Gly12Val)
KRAS	G12V	c.35G>T (p. Gly12Val) 42%
KRAS	G12V	c.35G>T (p. Gly12Val) 31%
KRAS	G12V	c.35G>T (p.Gly12Val) 3%
KRAS	G12V	c.35G>T (p.Gly12Val) 18%
KRAS	G12V	c.35G>T (p.Gly12Val) 15%
KRAS	G12V	c.35G>T (p.Gly12Val) 29%
KRAS	G12V	c.35G>T (p.Gly12Val) 29%
KRAS	G12V	c.35G>T (p.Gly12Val) 19%
KRAS	G12V	c.35G>T (p.Gly12Val) 54%
KRAS	G12V	c.35G>T (p.Gly12Val) 11%
KRAS	G12V	c.35G>T (p.Gly12Val) 28%
KRAS	G12V	c.35G>T (p.Gly12Val) 7%
KRAS	G12V	c.35G>T (p.Gly12Val)
KRAS	G12V	c.35G>T (p.Gly12Val) 10%
KRAS	G12V	c.35G>T (p. Gly12Val) 16%
KRAS	G12V	c.35G>T (p.Gly12Val) 19%
KRAS	G12V	c.35G>T (p.Gly12Val) 16%
KRAS	G12V	c.35G>T (p.Gly12Val) 10%
KRAS	G12V	c.35G>T (p.Gly12Val) 44%
KRAS	G12V	c.35G>T (p.Gly12Val) 20%
KRAS	G12V	c.35G>T (p. Gly12Val) 23%
KRAS	G12V	c.35G>T (p.Gly12Val)
KRAS	G12V	c.35G>T (p.Gly12Val) 37%
KRAS	G12V	c.35G>T (p.Gly12Val) 13%
KRAS	G12V	c.35G>T (p.Gly12Val) 20%
KRAS	G12V	c.35G>T (p.Gly12Val) 11%
KRAS	G12V	c.35G>T (p.Gly12Val) 6%
KRAS	G12V	c.35G>T (p.Gly12Val) 3%

KRAS	G12V	c.35G>T (p.Gly12Val) 17%
KRAS	G12V	c.35G>T (p. Gly12Val) 28%
KRAS	G12V	c.35G>T (p.Gly12Val) 36%
KRAS	G12V	c.35G>T (p.Gly12Val) 21%
KRAS	G12V	c.35G>T (p.Gly12Val) 33%
KRAS	G12V	c.35G>T (p.Gly12Val) 20%
KRAS	G12V	c.35G>T (p.Gly12Val)
KRAS	G12V	c.35G>T (p.Gly12Val)
KRAS	G12V	c.35G>T (p.Gly12Val) 31%
KRAS	G12V	c.35G>T (p.Gly12Val) 19%
KRAS	G12V	c.35G>T (p.Gly12Val) 33%
KRAS	G12V	c.35G>T (p.Gly12Val)
KRAS	G12V	c.35G>T (p.Gly12Val) 8%
KRAS	G12V	c.35G>T (p.Gly12Val) 7%
KRAS	G12V	c.35G>T (p.Gly12Val) 55%
KRAS	G12V	c.35G>T (p.Gly12Val) 5%
KRAS	G12V	c.35G>T (p. Gly12Val) 12%
KRAS	G12V	c.35G>T (p.Gly12Val) 16%
KRAS	G12V	c.35G>T (p.Gly12Val) 30%
KRAS	G12V	c.35G>T (p.Gly12Val) 6%
KRAS	G12V	c.35G>T (p.Gly12Val) 29%
KRAS	G12V	c.35G>T (p. Gly12Val) 14%
KRAS	G12V	c.35G>T (p.Gly12Val)
KRAS	G12V	c.35G>T (p.Gly12Val) 43%
KRAS	G12V	c.35G>T (p.Gly12Val) 22%
KRAS	G12V	c.35G>T (p.Gly12Val) 73%
KRAS	G12V	c.35G>T (p.Gly12Val) 29%
KRAS	G12V	c.35G>T (p.Gly12Val) 33%
KRAS	G12V	c.35G>T (p.Gly12Val) 41%
KRAS	G12V	c.35G>T (p.Gly12Val) 19%
KRAS	G12V	c.35G>T (p.Gly12Val) 8%
KRAS	G12V	c.35G>T (p.Gly12Val) 9%
KRAS	G12V	c.35G>T (p.Gly12Val)
KRAS	G12V	c.35G>T (p.Gly12Val)
KRAS	G12V	c.35G>T (p.Gly12Val) 3%
KRAS	G12V	c.35G>T (p.Gly12Val) 20%
KRAS	G12V	c.35G>T (p.Gly12Val) 12%
KRAS	G12V	c.35G>T (p.Gly12Val) 15%
KRAS	G12V	c.35G>T (p.Gly12Val) 18%
KRAS	G12V	c.35G>T (p.Gly12Val) 11%
KRAS	G12V	c.35G>T (p.Gly12Val) 22%
KRAS	G12V	c.35G>T (p.Gly12Val) 14%
KRAS	G12V	c.35G>T (p.Gly12Val) 35%
KRAS	G12V	c.35G>T (p.Gly12Val) 28%
KRAS	G12V	c.35G>T (p.Gly12Val) 4%
KRAS	G12V	c.35G>T (p.Gly12Val) 30%
KRAS	G12V	c.35G>T (p.Gly12Val) 16%

KRAS	G12V	c.35G>T (p.Gly12Val) 57%
KRAS	G12V	c.35G>T (p.Gly12Val) 21%
KRAS	G12V	c.35G>T (p.Gly12Val) 24%
KRAS	G12V	c.35G>T (p.Gly12Val) 5%
KRAS	G12V	c.34G>T (p.Gly12Val) 33%
KRAS	G12V	c.35G>T (p.Gly12Val) 29%
KRAS	G12V	c.35G>T (p.Gly12Val) 21%
KRAS	G12V	c.35G>T (p.Gly12Val) 20%
KRAS	G13C	c.37G>T (p.Gly13Cys) 27%
KRAS	G13C	c.37G>T (p.Gly13Cys) 68%
KRAS	G13C	c.37G>T (p.Gly13Cys) 19%
KRAS	G13C	c.37G>T (p.Gly13Cys) 34%
KRAS	G13C	c.37G>T (p.Gly13Cys) 28%
KRAS	G13C	c.37G>T (p.Gly13Cys)
KRAS	G13C	c.37G>T (p.Gly13Cys) 17%
KRAS	G13C	c.37G>T (p.Gly13Cys) 28%
KRAS	G13C	c.37G>T (p.Gly13Cys)
KRAS	G13C	c.37G>T (p.Gly13Cys) 81%
KRAS	G13C	c.36_37delTGinsGT (p.Gly13Cys) 18%
KRAS	G13C	c.37G>T (p.Gly13Cys) 30%
KRAS	G13D	c.38G>A (p.Gly13Asp) 49%
KRAS	G13D	c.38G>A (p.Gly13Asp)
KRAS	G13D	c.38_39delCGCinsAT (p.Gly13Asp) 74%
KRAS	G13D	c.38G>A (p.Gly13Asp) 22%
KRAS	G13D	c.38G>A (p.Gly13Asp)
KRAS	G13D	c.38G>A (p.Gly13Asp) 9%
KRAS	G13D	c.38G>A (p.Gly13Asp) 5%
KRAS	G13D	c.38G>A (p.Gly13Asp) 60%
KRAS	G13D	c.38G>A (p.Gly13Asp) 13%
KRAS	G13D	c.38G>A (p.Gly13Asp) 4%
KRAS	OTHER	c.34G>A (p.Gly12Ser) 14%
KRAS	OTHER	c.34G>A (p.Gly12Ser) 8%
KRAS	OTHER	c.436G>A (p.Ala146Thr) 42%
KRAS	OTHER	c.176C>G (p.Ala59Gly) 20%
KRAS	OTHER	c.34G>A (p.Gly12Ser) 89%
KRAS	OTHER	c.437C>T (p.Ala146Val) 24%
KRAS	OTHER	c.38_39delGCinsTG (p.Gly13Val) 57%
KRAS	OTHER	c.57G>C (p.Leu19Phe) 8%
KRAS	OTHER	c.34G>A (p.Gly12Ser) 37%
KRAS	OTHER	c.34G>A (p.Gly12Ser) 28%
KRAS	OTHER	c.35_37delGTGinsCCT (p.Gly12_Gly13delinsAlaCys) 42%
KRAS	OTHER	c.34_35delGGinsTC (p.Gly12Ser) 10%
KRAS	OTHER	c.34G>A (p.Gly12Ser) 34%
KRAS	OTHER	c.437C>T (p.Ala146Val) 45%
KRAS	OTHER	c.57G>T (p.Leu19Phe) 30%
KRAS	OTHER	c.34G>A (p.Gly12Ser) 87%
KRAS	Q61H	c.183A>C (p.Gln61His) 8%

KRAS	Q61H	c.183A>C (p.Gln61His) 15%
KRAS	Q61H	c.183A>T (p.Gln61His) 16%
KRAS	Q61H	c.183A>T (p.Gln61His) 78%
KRAS	Q61H	c.183A>T (p.Gln61His)
KRAS	Q61H	c.183A>T (p.Gln61His) 42%
KRAS	Q61H	c.183A>C (p.Gln61His) 31%
KRAS	Q61H	c.183A>C (p.Gln61His) 71%
KRAS	Q61H	c.183A>T (p.Gln61His) 15%
KRAS	Q61H	c.183A>C (p.Gln61His) 45%
KRAS	Q61H	c.183A>C (p.Gln61His) 56%
KRAS	Q61H	c.183A>C (p. Gln61His) 26%
KRAS	Q61H	c.183A>C (p.Gln61His) 21%
KRAS	Q61H	c.183A>C (p.Gln61His) 50%
KRAS	Q61H	c.183A>T (p.Gln61His) 26%
KRAS	Q61H	c.183A>T (p.Gln61His) 41%
KRAS	Q61H	c.183A>C (p.Gln61His)
KRAS	Q61H	c.183A>C (p.Gln61His)
KRAS	Q61H	c.183A>C (p.Gln61His) 9%
KRAS	Q61H	c.183A>T (p.Gln61His) 30%
KRAS	Q61H	c.183A>C (p.Gln61His) 25%
KRAS	Q61H	c.183A>T (p.Gln61His) 12%
KRAS	Q61H	c.183A>C (p.Gln61His) 40%
KRAS	Q61H	c.183A>T (p.Gln61His) 6%
KRAS	Q61H	c.183A>C (p.Gln61His) 24%
KRAS	Q61H	c.183A>T (p.Gln61His) 8%
KRAS	Q61H	c.183A>C (p.Gln61His) 13%
KRAS	Q61H	c.183A>T (p.Gln61His) 21%
KRAS	Q61H	c.183A>T (p.Gln61His) 32%
KRAS	Q61H	c.183A>C (p.Gln61His) 11%
KRAS	Q61L	c.182A>T (p.Gln61Leu) 6%
KRAS	Q61L	c.182A>T (p.Gln61Leu) 3.5%
KRAS	Q61L	c.182A>T (p.Gln61Leu) 42%
KRAS	Q61L	c.182A>T (p.Gln61Leu) 8%
KRAS	Q61L	C.182A>T (p.Gln61Leu) 22%
KRAS	Q61L	c.182A>T (p.Gln61Leu) 40%
KRAS	Q61L	c.182A>T (p.Gln61Leu) 39%
MET	EXON 14 SPLICE SITE	c.2942-29_2942-2del (p.?) 30%
MET	EXON 14 SPLICE SITE	c.3082+1G>T (p.?) 55%
MET	EXON 14 SKIPPING	Unknown
MET	NON-SPLICE SITE MISSENSE	c.2962C>T (p.Arg988Cys) 61%
NRAS	G12V	c.35G>T (p.Gly12Val) 32%
NRAS	G12V	c.35G>T (p.Gly12Val)
NRAS	Q61R	c.182A>G (p.Gln61Arg) 67%
PI3KCA	A990G	c.2969C>G (p.Ala990Gly) 43%
PI3KCA	D1029H	c.3085G>C (p.Asp1029His) 12%
PI3KCA	E542K	c.1624G>A (p.Glu542Lys) 13%
PI3KCA	E542K	c.1624G>A (p. Glu542Lys) 28%

PI3KCA	E542K	c.1624G>A (p.Glu542Lys) 65%
PI3KCA	E542K	c.1624G>A (p.Glu542Lys) 9%
PI3KCA	E545G	c.1634A>G (p.Glu545Gly) 24%
PI3KCA	E545K	c.1633G>A (p.Glu545Lys) 6%
PI3KCA	E545K	Unknown
PI3KCA	E545K	c.1633G>A (p.Glu545Lys)
PI3KCA	E545K	c.1633G>A (p.Glu545Lys) 19%
PI3KCA	E545K	c.1633G>A (p.Glu545Lys) 12%
PI3KCA	E545K	c.1633G>A (p.Glu545Lys) 10%
PI3KCA	E545K	c.1633G>A (p.Glu545Lys) 5%
PI3KCA	E545K	c.1633G>A (p. Glu545Lys) 14%
PI3KCA	E545K	c.1633G>A (p.Glu545Lys) 31%
PI3KCA	E545K	c.1633G>A (p.Glu545Lys) 11%
PI3KCA	E545K	c.1633G>A (p. Glu545Lys) 23%
PI3KCA	E545K	c.1633G>A (p.Glu545Lys) 14%
PI3KCA	E545K	c.1633G>A (p.Glu545Lys) 21%
PI3KCA	E545K	c.1633G>A (p.Glu545Lys) 18%
PI3KCA	E545K	c.1633G>A (p.Glu545Lys) 9%
PI3KCA	E545N	c.1633G>C (p.Glu545Gln) 11%
PI3KCA	H1047L	c.3140A>T (p.His1047Leu) 10%
PI3KCA	H1047L	c.3140A>T (p.His1047Leu) 46%
PI3KCA	H1047L	c.3140A>T (p.His1047Leu) 27%
PI3KCA	H1047L	c.3140A>T (p.His1047Leu) 23%
PI3KCA	H1047L	c.3140A>T (p.His1047Leu) 26%
PI3KCA	H1047L	c.3140A>T (p.His1047Leu) 14%
PI3KCA	H1047R	c.3140A>G (p.His1047Arg) 3%
PI3KCA	H1047R	c.3140A>G (p.His1047Arg) 18%
PI3KCA	H1047R	c.3140A>G (p.His1047Arg) 21%
PI3KCA	H1047R	c.3140A>G (p.His1047Arg) 19%
PI3KCA	H1047R	c.3140A>G (p.His1047Arg) 12%
PI3KCA	H1047R	c.3140A>G (p.His1047Arg) 15%
PI3KCA	H1047R	c.3140A>G (p.His1047Arg) 6%
PI3KCA	H1047R	c.3140A>G (p.His1047Arg) 44%
PI3KCA	H1047R	c.3140A>G (p.His1047Arg) 10%
PI3KCA	H1048R	c.3143A>G (p.His1048Arg) 24%
PI3KCA	N1044K	c.3132T>A (p.Asn1044Lys) 24%
PI3KCA	N345L	c.1035T>A (p.Asn345Lys) 5%
PI3KCA	N515S	c.1544A>G (p.Asn515Ser) 51%
PI3KCA	P539R	c.1616C>G (p.Pro539Arg) 6%
PI3KCA	Q546K	c.1636C>A (p.Gln546Lys) 8%
PI3KCA	T1021H	c.3061T>C (p. Tyr1021His) 34%
PI3KCA	T1025S	c.3073A>T (p.Thr1025Ser) 33%
RET	NON-SENSE	c.2778dupT (p. lle927Tyrfs*7) 11%
RET	SPLICE SITE VARIANT	c.2801+2T>C (p.?) 10%
TP53	DISRUPTIVE NON-FUNCTIONAL	c.743G>A (p.Arg248Gln) 29%
TP53	DISRUPTIVE NOS	c.177dupT (p.Pro60Serfs*3) 58%
TP53	DISRUPTIVE, FRAMESHIFT	c.401_402insG (p.Phe134Leufs*15) 52%

TP53	DISRUPTIVE, FRAMESHIFT	c.432delG (p.Gln144Hisfs*26) 21%
TP53	DISRUPTIVE, FRAMESHIFT	c.1008_1020delGCGCTTCGAGATG (p.Glu336Aspfs*5) 35%
TP53	DISRUPTIVE, FRAMESHIFT	c.902dupC (p.Gly302Argfs*4) 15%
TP53	DISRUPTIVE, FRAMESHIFT	c.626_627delGA (p.Arg209Lysfs*6) 19%
TP53	DISRUPTIVE, FRAMESHIFT	c.298delC (p.Gln100Argfs*23) 20%
TP53	DISRUPTIVE, FRAMESHIFT	c.880_881insT (p.Glu294Valfs*12) 8%
TP53	DISRUPTIVE, FRAMESHIFT	c.1045delG (p.Glu349Asnfs*21) 17%
TP53	DISRUPTIVE, FRAMESHIFT	c.105_111delGCCGTCC (p.Leu35Phefs*7) 25%
TP53	DISRUPTIVE, FRAMESHIFT	c.454_466delCCGCCCGGCACCC (p.Pro152Alafs*14) (37%)
TP53	DISRUPTIVE, FRAMESHIFT	c.526_528delinsGG (p.Cys176Glyfs*71) 37%
TP53	DISRUPTIVE, FRAMESHIFT	c.1124_1127delAGTC (p.Gln375Leufs*46) 22%
TP53	DISRUPTIVE, FRAMESHIFT	c.603delG (p.Leu201Phefs*46) 38%
TP53	DISRUPTIVE, FRAMESHIFT	c.716delA (p.Asn239Thrfs*8) 95%
TP53	DISRUPTIVE, FRAMESHIFT	c.532delC (p.His178Thrfs*69) 38%
TP53	DISRUPTIVE, FRAMESHIFT	c.168_169delAGinsT, (p.Glu56Aspfs*67) 10%
TP53	DISRUPTIVE, FRAMESHIFT	c.703_705delinsT (p.Asn235Leufs*4) 32%
TP53	DISRUPTIVE, FRAMESHIFT	c.41_42delinsCCCTCA (p.Leu14Profs*16) 20%
TP53	DISRUPTIVE, FRAMESHIFT	c.220delG (p.Ala74Profs*49) 23%
TP53	DISRUPTIVE, FRAMESHIFT	c.949delC (p.Gln317Serfs*28) 27%
TP53	DISRUPTIVE, FRAMESHIFT	c.1176dupA (p.Asp393Argfs*78) 50%
TP53	DISRUPTIVE, FRAMESHIFT	c.717_723delCAGTTCCinsGT (p.Asn239Lysfs*23) 35%
TP53	DISRUPTIVE, FRAMESHIFT	c.715_716delAA (p.Asn239Glnfs*24) 78%
TP53	DISRUPTIVE, FRAMESHIFT	c.848_849delGC (p.Arg283Hisfs*22) 6%
TP53	DISRUPTIVE, FRAMESHIFT	c.120delC (p.Pro47Argfs*76) 40%
TP53	DISRUPTIVE, FRAMESHIFT	c.88_89delAA (p.Asn30Argfs*12) 34%
TP53	DISRUPTIVE, FRAMESHIFT	c.102delC (p.Leu35Cysfs*9) 16%
TP53	DISRUPTIVE, FRAMESHIFT	c.1118delA (p.Lys373Argfs*49) (52%)
TP53	DISRUPTIVE, FUNCTIONAL	c.566C>G (p.Ala189Gly) 58%
TP53	DISRUPTIVE, GAIN OF FUNCTION	c.742C>T (p.Arg248Trp) 8%
TP53	DISRUPTIVE, GAIN OF FUNCTION	c.527G>T (p.Cys176Phe) 10%
TP53	DISRUPTIVE, GAIN OF FUNCTION	c.743G>A (p.Arg248Gln) 52%
TP53	DISRUPTIVE, GAIN OF FUNCTION	c.559G>A (p.Gly187Ser) 12%
TP53	DISRUPTIVE, GAIN OF FUNCTION	c.538G>A (p.Glu180Lys) 60%
TP53	DISRUPTIVE, GAIN OF FUNCTION	c.536A>T (p.His179Leu) 20%
TP53	DISRUPTIVE, GAIN OF FUNCTION	c.742C>T (p.Arg248Trp) 4%
TP53	DISRUPTIVE, GAIN OF FUNCTION	c.743G>A (p.Arg248Gln) 49%
TP53	DISRUPTIVE, GAIN OF FUNCTION	c743G>A (p.Arg248Gln) 13%
TP53	DISRUPTIVE, MISSENSE	c.313G>T (p.Gly105Cys)
TP53	DISRUPTIVE, NONFUNCTIONAL	c.733G>C (p.Gly245Arg) 12%
TP53	DISRUPTIVE, NONFUNCTIONAL	c.706T>C (p.Tyr236His) 15%
TP53	DISRUPTIVE, NONFUNCTIONAL	c.581T>G (p.Leu194Arg) 40%
TP53	DISRUPTIVE, NON-FUNCTIONAL	c.743G>T (p.Arg248Leu) 38%
TP53	DISRUPTIVE, NON-FUNCTIONAL	c.799C>T (p.Arg267Trp) 54%
TP53	DISRUPTIVE, NON-FUNCTIONAL	c.728T>C (p.Met243Thr) 10%
TP53	DISRUPTIVE, NON-FUNCTIONAL	c.710T>A (p.Met237Lys) 31%
TP53	DISRUPTIVE, NON-FUNCTIONAL	c.743_744delGGinsTT (p.Arg248Leu) 6%
TP53	DISRUPTIVE, NON-FUNCTIONAL	c.715A>G (p.Asn239Asp) 59%

TP53	DISRUPTIVE, NON-FUNCTIONAL	c.584T>C (p.Ile195Thr) 55%
TP53	DISRUPTIVE, NON-FUNCTIONAL	c.724T>G (p.Cys242Gly) 33%
TP53	DISRUPTIVE, NON-FUNCTIONAL	c.584T>C (p.Ile195Thr) 19%
TP53	DISRUPTIVE, NON-FUNCTIONAL	c.733G>T (p.Gly245Cys) 69%
TP53	DISRUPTIVE, NON-FUNCTIONAL	c.733G>T (p.Gly245Cys) 82%
TP53	DISRUPTIVE, NON-FUNCTIONAL	c.518T>A (p.Val173Glu) 28%
TP53	DISRUPTIVE, NON-FUNCTIONAL	c.734G>T (p.Gly245Val) 34%
TP53	DISRUPTIVE, NON-FUNCTIONAL	c.1025G>C (p.Arg342Pro) 24%
TP53	DISRUPTIVE, NON-FUNCTIONAL	c.743G>T (p.Arg248Leu) 13%
TP53	DISRUPTIVE, NON-FUNCTIONAL	c.526T>C (p.Cys176Arg) 41%
TP53	DISRUPTIVE, NON-FUNCTIONAL	c.742C>G (p.Arg248Gly) 19%
TP53	DISRUPTIVE, NON-FUNCTIONAL	c.722C>T (p.Ser241Phe) 5%
TP53	DISRUPTIVE, NON-FUNCTIONAL	c.581T>G (p.Leu194Arg) 61%
TP53	DISRUPTIVE, NON-FUNCTIONAL	c.734G>T (p.Gly245Val) 27%
TP53	DISRUPTIVE, NON-FUNCTIONAL	c.745A>T (p.Arg249Trp) 79%
TP53	DISRUPTIVE, NON-FUNCTIONAL	c.737T>A (p.Met246Lys) 59%
TP53	DISRUPTIVE, NON-FUNCTIONAL	c.743_744delGGinsTT (p.Arg248Leu) 64%
TP53	DISRUPTIVE, NON-FUNCTIONAL	c.523C>G (p.Arg175Gly) 35%
TP53	DISRUPTIVE, NON-FUNCTIONAL	c.584T>C (p.Ile195Thr) 87%
TP53	DISRUPTIVE, NON-FUNCTIONAL	c.743G>C (p.Arg248Pro) 12%
TP53	DISRUPTIVE, NON-FUNCTIONAL	c.584T>C (p.Ile195Thr) 40%
TP53	DISRUPTIVE, NON-FUNCTIONAL	c.537T>A (p.His179Gln) 11%
TP53	DISRUPTIVE, NON-SENSE	c.796T>G (p.Gly266*) 96%
TP53	DISRUPTIVE, NON-SENSE	c.376-1G>A (p.?) 20%
TP53	DISRUPTIVE, NON-SENSE	c.747G>T (p.Arg249Ser) 43%
TP53	DISRUPTIVE, NON-SENSE	c.574C>T (p.Gln192*)
TP53	DISRUPTIVE, NON-SENSE	c.742C>G (p.Arg248Gly) 29%
TP53	DISRUPTIVE, NON-SENSE	c.142dupG (p.Asp48Glyfs*4) 85%
TP53	DISRUPTIVE, NON-SENSE	c.742C>T (p.Arg248Trp) 95%
TP53	DISRUPTIVE, NON-SENSE	c.949C>T (p.Gln317*)
TP53	DISRUPTIVE, NON-SENSE	c.934_935delAC (p.Thr312GLNfs*24) 43%
TP53	DISRUPTIVE, NON-SENSE	c.818G>C (p. Arg273Pro) 15%
TP53	DISRUPTIVE, NON-SENSE	c.853G>T (p.Glu285*)
TP53	DISRUPTIVE, NON-SENSE	c.425_430delCTGTGC (p.Pro142_Val143del)
TP53	DISRUPTIVE, NON-SENSE	c.617dupT (Leu206Phefs*3) 21%
TP53	DISRUPTIVE, NON-SENSE	c.734G>A (p.Gly245Asp)
TP53	DISRUPTIVE, NON-SENSE	c.991C>T (p.Gln331*) 61%
TP53	DISRUPTIVE, NON-SENSE	c.559+2T>C (p. ?) 93%
TP53	DISRUPTIVE, NON-SENSE	c.321C>G (p.Tyr107*) 10%
TP53	DISRUPTIVE, NON-SENSE	c.800G>C (p. Arg267Pro) 42%
TP53	DISRUPTIVE, NON-SENSE	c.471_472insT (p.Arg158Serfs*23)
TP53	DISRUPTIVE, NON-SENSE	c.717C>G (p.Asn239Lys) 48%
TP53	DISRUPTIVE, NON-SENSE	c.796G>T (p.Gly266*)
TP53	DISRUPTIVE, NON-SENSE	c.592G>T (p.Glu198*) 44%
TP53	DISRUPTIVE, NON-SENSE	c.880G>T (p.Glu294*) 33%
TP53	DISRUPTIVE, NON-SENSE	c.871A>T (p.Lys291*) K291*
TP53	DISRUPTIVE, NON-SENSE	c.712_713insTT (p.Cys238Phefs*10)

TP53	DISRUPTIVE, NON-SENSE	c.375+1G>T (p.?)
TP53	DISRUPTIVE, NON-SENSE	c.535C>T (p.His179Tyr)
TP53	DISRUPTIVE, NON-SENSE	c.273G>A (p.Trp91*) 32%
TP53	DISRUPTIVE, NON-SENSE	c.673-1G>T (p.?) 68%
TP53	DISRUPTIVE, NON-SENSE	c.586C>T (p.Arg196*) 53%
TP53	DISRUPTIVE, NON-SENSE	c.743G>A (p.Arg248Gln) 47%
TP53	DISRUPTIVE, NON-SENSE	c.112C>T (p.Gln38*) 21%
TP53	DISRUPTIVE, NON-SENSE	c.725G>T (p.Cys242Phe) 23%
TP53	DISRUPTIVE, NON-SENSE	c.916C>T (p. Arg306*) 39%
TP53	DISRUPTIVE, NON-SENSE	c.586C>T (p.Arg196*) 23%
TP53	DISRUPTIVE, NON-SENSE	c.731G>A (p.Gly244Asp) 51%
TP53	DISRUPTIVE, NON-SENSE	c.461delG (p. Gly154Alafs*16) 67%
TP53	DISRUPTIVE, NON-SENSE	c.569delC (p. Pro190Leufs*57) 56%
TP53	DISRUPTIVE, NON-SENSE	c.584T>C (p.Ile195Thr) 15%
TP53	DISRUPTIVE, NON-SENSE	c.902delC (p.Pro301Glnfs*44) 9%
TP53	DISRUPTIVE, NON-SENSE	c.673-1G>T (p.?) 18%
TP53	DISRUPTIVE, NON-SENSE	c.637_639delCGAinsTGG (p.Arg213Trp) 20%
TP53	DISRUPTIVE, NON-SENSE	c.726C>A (p.Cys242*) 59%
TP53	DISRUPTIVE, NON-SENSE	c.267delC (p.Ser90Profs*33) 31%
TP53	DISRUPTIVE, NON-SENSE	c.991C>T (p.Gln331*) 13%
TP53	DISRUPTIVE, NON-SENSE	c.892G>T (p.Glu298*) 38%
TP53	DISRUPTIVE, NON-SENSE	c.112C>T (p.Gln38*) 36%
TP53	DISRUPTIVE, NON-SENSE	c.863delA (p.Asn288Ilefs*57) 64%
TP53	DISRUPTIVE, NON-SENSE	c.475_476delGC (p.Ala159Hisfs*21) 16%
TP53	DISRUPTIVE, NON-SENSE	c.493C>T (p.Gln165*) 44%
TP53	DISRUPTIVE, NON-SENSE	c.673-2A>G (p.?) 67%
TP53	DISRUPTIVE, NON-SENSE	c.375+1G>A (p.?) 12%
TP53	DISRUPTIVE, NON-SENSE	c.375G>T (p.Thr125=) 10%
TP53	DISRUPTIVE, NON-SENSE	c.586C>T (p.Arg196*)
TP53	DISRUPTIVE, NON-SENSE	c.418_419insTA (p.Thr140Ilefs*31) 21%
TP53	DISRUPTIVE, NON-SENSE	c.559+1G>A (p.?) 57%
TP53	DISRUPTIVE, NON-SENSE	c.919+1G>T (p.?) 27%
TP53	DISRUPTIVE, NON-SENSE	c.586C>T (p.Arg196*) 84%
TP53	DISRUPTIVE, NON-SENSE	c.216dupC (p.Val173Argfs*76) 63%
TP53	DISRUPTIVE, NON-SENSE	c.154C>T (p.Gln52*) 28%
TP53	DISRUPTIVE, NON-SENSE	c.725G>T (p.Cys242Phe) 44%
TP53	DISRUPTIVE, NON-SENSE	c.576_578delGCA (p.Gln192del)
TP53	DISRUPTIVE, NON-SENSE	c.535C>T (p.His179Tyr) 8%
TP53	DISRUPTIVE, NON-SENSE	c.267delC (p.Ser90Profs*33) 17%
TP53	DISRUPTIVE, NON-SENSE	c.375G>T (p.Thr125=) 34%
TP53	DISRUPTIVE, NON-SENSE	c.1006G>T (p.Glu336*) 7%
TP53	DISRUPTIVE, NON-SENSE	c. 856G>C (p.Glu286Gln) 52%
TP53	DISRUPTIVE, NON-SENSE	c.988delC (p.Leu330Phefs*15)
TP53	DISRUPTIVE, NON-SENSE	c.993+1G>T 14%
TP53	DISRUPTIVE, NON-SENSE	c.548C>G (p.Ser183*) 15%
TP53	DISRUPTIVE, NON-SENSE	c.586C>T (p.Arg196*) 23%
TP53	DISRUPTIVE, NON-SENSE	c.920-1G>T (p.?)

TP53	DISRUPTIVE, NON-SENSE	c.586C>T (p.Arg196*)
TP53	DISRUPTIVE, NON-SENSE	c.995T>A (p. Ile332Asn) 94%
TP53	DISRUPTIVE, NON-SENSE	c.408delA (p.Gln136Hisfs*34)
TP53	DISRUPTIVE, NON-SENSE	c.515T>A (p.Val172Asp) 19%
TP53	DISRUPTIVE, NON-SENSE	c.955A>T (p.Lys319*) 19%
TP53	DISRUPTIVE, NON-SENSE	c.818G>T (p.Arg273Leu) 10%
TP53	DISRUPTIVE, NON-SENSE	c.991C>T (p.Gln331*) 18%
TP53	DISRUPTIVE, NON-SENSE	c.880G>T (p.Glu294*) 16%
TP53	DISRUPTIVE, NON-SENSE	c.722_723delinsT (p.Ser241Phefs*6) 29%
TP53	DISRUPTIVE, NON-SENSE	c.592G>T (p.Glu198*)
TP53	DISRUPTIVE, NON-SENSE	c.375G>T (p.Thr125=) (14%)
TP53	DISRUPTIVE, NON-SENSE	c.610dupG (p.Glu204Glyfs*5) 25%
TP53	DISRUPTIVE, NON-SENSE	c.499C>T (p.Gln167*) 34%
TP53	DISRUPTIVE, NON-SENSE	c.746G>C (p.Arg249Thr) 15%
TP53	DISRUPTIVE, NON-SENSE	c.455delC (p.Pro152Argfs*18) 6%
TP53	DISRUPTIVE, NON-SENSE	c.745A>G (p.Arg249Gly) 18%
TP53	DISRUPTIVE, NON-SENSE	c.586C>T (p.Arg196*) 41%
TP53	DISRUPTIVE, NON-SENSE	c.734G>A (p.Gly245Asp)
TP53	DISRUPTIVE, NON-SENSE	c.949C>T (p.Gln317*)
TP53	DISRUPTIVE, NON-SENSE	c.796G>T (p.Gly266*) 70%
TP53	DISRUPTIVE, NON-SENSE	c.152_158delAACAATG (p.Glu51Glyfs*70)
TP53	DISRUPTIVE, NON-SENSE	c.102dupC (p.Pro36Alafs*7)
TP53	DISRUPTIVE, NON-SENSE	c.281C>G (p.Ser94*) 69%
TP53	DISRUPTIVE, NON-SENSE	c.725G>T (p.Cys242Phe) 69%
TP53	DISRUPTIVE, NON-SENSE	c.711_713delGTGinsTTT (p.Met237_Cys238delinsIlePhe) 49%
TP53	DISRUPTIVE, NON-SENSE	c.522G>T (p.Arg174Ser) 57%
TP53	DISRUPTIVE, NON-SENSE	c.637C>T (p.Arg213*) 81%
TP53	DISRUPTIVE, NON-SENSE	c.560-1G>T (p.?)
TP53	DISRUPTIVE, NON-SENSE	c.874A>T (p.Lys292*)
TP53	DISRUPTIVE, NON-SENSE	c.1006G>T (p.Glu336*) 58%
TP53	DISRUPTIVE, NON-SENSE	c.718A>G (p. Ser240Gly) 11%
TP53	DISRUPTIVE, NON-SENSE	c.797G>A (p.Gly266Glu) 23%
TP53	DISRUPTIVE, NON-SENSE	c.831T>A (p.Cys277*) 31%
TP53	DISRUPTIVE, NON-SENSE	c.672+1G>T (p.?) 65%
TP53	DISRUPTIVE, NON-SENSE	c.592delG (p.Glu198Lysfs*49) 14%
TP53	DISRUPTIVE, NON-SENSE	c.202G>T (p.Glu68*) 26%
TP53	DISRUPTIVE, NON-SENSE	c.610G>T (p.Glu204*) 16%
TP53	DISRUPTIVE, NON-SENSE	c.825_832delTGCCTGTCinsAGGTG (p.Cys275*) 87%
TP53	DISRUPTIVE, NON-SENSE	c.614A>G (p.Tyr205Cys) 15%
TP53	DISRUPTIVE, NON-SENSE	c.721T>G (p.Ser241Ala) 43%
TP53	DISRUPTIVE, NON-SENSE	c.742C>T (p.Arg248Trp) 54%
TP53	DISRUPTIVE, NON-SENSE	c.459delC (p.Gly154Alafs*16) 29%
TP53	DISRUPTIVE, NON-SENSE	c.1024C>T (p.Arg342*) 50%
TP53	DISRUPTIVE, NON-SENSE	c.314dupG (p.Ser106Glnfs*43) 41%
TP53	DISRUPTIVE, NON-SENSE	c.535C>T (p.His179Tyr) 47%
TP53	DISRUPTIVE, NON-SENSE	c.602T>A (p.Leu201*) 23%
TP53	DISRUPTIVE, NON-SENSE	c.673-2A>C (p?) 19%

TP53	DISRUPTIVE, NON-SENSE	c.326delT (p.Phe109Serfs*14) 37%
TP53	DISRUPTIVE, NON-SENSE	c.839G>T (p.Arg280Ile) 71%
TP53	DISRUPTIVE, NON-SENSE	c.365_366delTG (p.Val122Aspfs*26)
TP53	DISRUPTIVE, NON-SENSE	c.811G>T (p.Glu271*) 11%
TP53	DISRUPTIVE, NON-SENSE	c.811G>T (p.Glu21*) 11%
TP53	DISRUPTIVE, NON-SENSE	c.96+2T>G (p.?)
TP53	DISRUPTIVE, NON-SENSE	c.1045G>T (p.Glu349*) 70%
TP53	DISRUPTIVE, NON-SENSE	c.574C>T (p.Gln192*)
TP53	DISRUPTIVE, NON-SENSE	c.529C>T (p.Pro177Ser) 16%
TP53	DISRUPTIVE, NON-SENSE	c.554_559delinsT (p.Ser185Metfs*22) 81%
TP53	DISRUPTIVE, NON-SENSE	c.1045G>T (p.Glu349*)
TP53	DISRUPTIVE, NON-SENSE	c.747_748delGCinsTT (p.Arg249_Pro250delinsSerSer) 58%
TP53	DISRUPTIVE, NON-SENSE	c.560-2A>T (p.?)
TP53	DISRUPTIVE, NON-SENSE	c.919+1G>T (p.?)
TP53	DISRUPTIVE, NON-SENSE	c.734G>T (p.Gly245Val)
TP53	DISRUPTIVE, NON-SENSE	c.859delG (p.Glu287Argfs*58) 31%
TP53	DISRUPTIVE, NON-SENSE	c.270delC (p.Trp91Glyfs*32)
TP53	DISRUPTIVE, NON-SENSE	c.97-2A>G (p.?)
TP53	DISRUPTIVE, NON-SENSE	c.742C>T (p.Arg248Trp) 21%
TP53	DISRUPTIVE, NON-SENSE	c782_782+14delGGTCAGGAGCCACTTdelinsCTG (p.Ser261Thrfs*85) 61%
TP53	DISRUPTIVE, NON-SENSE	c.490A>T (p.Lys164*) 30%
TP53	DISRUPTIVE, NON-SENSE	c.542G>C (p.Arg181Pro)
TP53	DISRUPTIVE, NON-SENSE	c.303_307delinsC (p.Lys101Asnfs*21)
TP53	DISRUPTIVE, NON-SENSE	c.796G>T (p.Gly266*)
TP53	DISRUPTIVE, NON-SENSE	c.602dupT (p.Leu201Phefs*8)
TP53	DISRUPTIVE, NON-SENSE	c.637C>T (p.Arg213*) 21%
TP53	DISRUPTIVE, NON-SENSE	c.772G>T (p.Glu258*) 16%
TP53	DISRUPTIVE, NON-SENSE	c.991C>T (p.Gln331*) 17%
TP53	DISRUPTIVE, NON-SENSE	c.880G>T (p.Glu294*) 65%
TP53	DISRUPTIVE, NON-SENSE	c.991C>T (p.Gln331*) 20%
TP53	DISRUPTIVE, NON-SENSE	c.586C>T (p.Arg196*) 82%
TP53	DISRUPTIVE, NON-SENSE	c.637C>T (p.Arg213*) 32%
TP53	DISRUPTIVE, NON-SENSE	c.1036G>T (p.Glu346*) 17%
TP53	DISRUPTIVE, NON-SENSE	c.158G>A (p.Trp53*) 34%
TP53	DISRUPTIVE, NON-SENSE	c.916C>T (p.Arg306*) 30%
TP53	DISRUPTIVE, NON-SENSE	c.637C>T (p.Arg213*) 71%
TP53	DISRUPTIVE, NON-SENSE	c.725G>T (p.Cys242Phe) 6%
TP53	DISRUPTIVE, NON-SENSE	c.892G>T (p.Glu298*) 20%
TP53	DISRUPTIVE, NON-SENSE	c.437G>A (p.Trp146*) 41%
TP53	DISRUPTIVE, NON-SENSE	c.637C>T (p.Arg213*) 39%
TP53	DISRUPTIVE, NON-SENSE	c.461delG (p.Gly154Alafs*16) 48%
TP53	DISRUPTIVE, NON-SENSE	c.743G>C (p. Arg248Pro) 30%
TP53	DISRUPTIVE, NON-SENSE	c.994-1G>T (p.?) 10%
TP53	DISRUPTIVE, NON-SENSE	c.571_577delCCTCAGC (p.Pro191Ilefs*54)
TP53	DISRUPTIVE, NON-SENSE	c.541C>T (p.Arg181Cys)
TP53	DISRUPTIVE, NON-SENSE	c.375G>T (p.Thr125=)
TP53	DISRUPTIVE, NON-SENSE	c.743G>A (p.Arg248Gln)

TP53	DISRUPTIVE, NON-SENSE	c.560-1G>A (p. ?) 20%
TP53	DISRUPTIVE, NON-SENSE	c.637C>T (p.Arg213*) 45%
TP53	DISRUPTIVE, NON-SENSE	c.1022dupT (p. Arg342Profs*5) 62%
TP53	DISRUPTIVE, NOS	c.870_873delCAAG (p.Lys292Glyfs*52) 16%
TP53	DISRUPTIVE, NOS	c.916C>T (p.Arg306*) 9%
TP53	DISRUPTIVE, NOS	c.783-1G>T (p.?) 42%
TP53	DISRUPTIVE, NOS	c.752T>G (p.Ile251Ser) 32%
TP53	DISRUPTIVE, NOS	c.705delC (p.Tyr236Thrfs*11) 57%
TP53	DISRUPTIVE, NOS	c.592G>T (p.Glu198*) 50%
TP53	DISRUPTIVE, NOS	c.673_2A>G (p.?) 10%
TP53	DISRUPTIVE, NOS	c.880_892delGAGCCTCACCACG (p.Glu294Serfs*47) 47%
TP53	DISRUPTIVE, NOS	c.294_297delTTCC (p.Ser99Argfs*23) 23%
TP53	DISRUPTIVE, NOS	c.473delG (p.Arg158Profs*12) 56%
TP53	DISRUPTIVE, NOS	c.375G>T (p.?) 22%
TP53	DISRUPTIVE, NOS	c.1015G>T (p.Glu339*) 26%
TP53	DISRUPTIVE, NOS	c.920-2A>G (p.?) 19%
TP53	DISRUPTIVE, NOS	c.338_348delTCTTGCATTCT (p.Phe113Trpfs*32) 57%
TP53	DISRUPTIVE, NOS	c.660T>G (p.Tyr220*) 24%
TP53	DISRUPTIVE, NOS	c.455dupC (p.Pro153Alafs*28) 31%
TP53	DISRUPTIVE, NOS	c.747_748delGCinsTA (p.Arg249_Pro250delinsSerThr) 33%
TP53	DISRUPTIVE, NOS	c.920-2A>T (p.?) 10%
TP53	DISRUPTIVE, NOS	c.782+1G>T (p.?) 13%
TP53	DISRUPTIVE, NOS	c.740A>T (p.Asn247Ile) 29%
TP53	DISRUPTIVE, NOS	c.920-11_927delCTCTTTCCTAGCACTGCCC (p.?) 12%
TP53	DISRUPTIVE, NOS	c.672+2T>A (p.?) 19%
TP53	DISRUPTIVE, NOS	c.920-2A>G (p.?) 25%
TP53	DISRUPTIVE, NOS	c.787_790delAATC (p.Asn263Tyrfs*81) 72%
TP53	DISRUPTIVE, NOS	c.919_919+1delinsAT (p.?) 16%
TP53	DISRUPTIVE, NOS	c.439delG (p.Val147Leufs*23) 15%
TP53	DISRUPTIVE, NOS	c.919+2T>A (p.?) 9%
TP53	DISRUPTIVE, NOS	c.96+1G>A (p.?) 38%
TP53	DISRUPTIVE, NOS	c.948_957delCCAGCCAAAG (p.Gln317Argfs*25) 74%
TP53	DISRUPTIVE, NOS	c.430C>T (p.Gln144*) 93%
TP53	DISRUPTIVE, NOS	c.734G>A (p.Gly245Asp) 28%
TP53	DISRUPTIVE, NOS	c.734G>A (p.Gly245Asp) 32%
TP53	DISRUPTIVE, NOS	c.714_715delTAinsGT (p.Cys238_Asn239delinsTrpTyr) 20%
TP53	DISRUPTIVE, NOS	c.783-2A>G (p.?) 60%
TP53	DISRUPTIVE, NOS	c.375+1G>T (p.?) 64%
TP53	DISRUPTIVE, NOS	c.797delG (p.Gly266Aspfs*79) 29%
TP53	DISRUPTIVE, NOS	c.916C>T (p.Arg306*) 26%
TP53	DISRUPTIVE, NOS	c.920-2A>C (p.?) 50%
TP53	DISRUPTIVE, NOS	c.376-2A>T (p.?) 39%
TP53	DISRUPTIVE, NOS	c.376-2A>G (p.?) 30%
TP53	DISRUPTIVE, NOS	c.473_490delGCGCCATGGCCATCTACA (p.Arg158_Lys164delinsGln) 32%
TP53	DISRUPTIVE, NOS	c.559+1G>T (p.?) 18%
TP53	DISRUPTIVE, NOS	c.118_130dupATGGATGATTTGA (p.Met44Asnfs*3) 35%
TP53	DISRUPTIVE, NOS	c.994-1G>C (p.?) 17%

TP53	DISRUPTIVE, NOS	c.783-1G>T (p.?) 41%
TP53	DISRUPTIVE, NOS	c.625A>T (p.Arg209*) 79%
TP53	DISRUPTIVE, NOS	c.642_643delTA (p.His214Glnfs*7) 7%
TP53	DISRUPTIVE, NOS	c.586C>T (p.Arg196*) 27%
TP53	DISRUPTIVE, NOS	c.375G>T (p.Thr125=) 22%
TP53	DISRUPTIVE, NOS	c.310C>T (p.Gln104*) 16%
TP53	DISRUPTIVE, NOS	c.560-1G>T (p.?) 53%
TP53	DISRUPTIVE, NOS	c.375+1delG (p.?) 33%
TP53	DISRUPTIVE, NOS	c.757A>G (p.Thr253Ala) 22%
TP53	DISRUPTIVE, NOS	c.515_522dupTTGTGAGG (p.Arg175Leufs*2) 20%
TP53	DISRUPTIVE, NOS	c.782+1delG (p.?) 79%
TP53	DISRUPTIVE, NOS	c.741_750delinsA p.(Asn247_Pro250delinsLys) 16%
TP53	DISRUPTIVE, NOS	c.920-6_921delTCCTAGCA (p.?) 36%
TP53	DISRUPTIVE, NOS	c.993+1G>T (p.?) 48%
TP53	DISRUPTIVE, NOS	c.393_399delCAAGATG (p.Lys132Phefs*36) 12%
TP53	DISRUPTIVE, NOS	c.981T>A (p.Tyr327*) 9%
TP53	DISRUPTIVE, PARTIALLY FUNCTIONAL	c.527G>T (p.Cys176Phe) 95%
TP53	DISRUPTIVE, PARTIALLY FUNCTIONAL	c.535C>T (p.His179Tyr) 90%
TP53	DISRUPTIVE, PARTIALLY FUNCTIONAL	c.542G>A (p.Arg181His) 12%
TP53	DISRUPTIVE, PARTIALLY FUNCTIONAL	c.538G>A (p.Glu180Lys) 81%
TP53	DISRUPTIVE, PARTIALLY FUNCTIONAL	c.527G>T (p.Cys176Phe) 20%
TP53	DISRUPTIVE, PARTIALLY FUNCTIONAL	c.535C>T (p.His179Tyr) 97%
TP53	DISRUPTIVE, SPLICE SITE	c.920-1G>T (p.?) 37%
TP53	DISRUPTIVE, SPLICE SITE	c.920-1G>T (p.?) 11%
TP53	DISRUPTIVE, SPLICE SITE	c.994-1G>T (p.?) 16%
TP53	DISRUPTIVE, SPLICE SITE	c.375G>T (p.Thr125=) 5%
TP53	DISRUPTIVE, SPLICE SITE	c.375G>T (p.Thr125=) 23%
TP53	DISRUPTIVE, SPLICE SITE	c.993+1G>T (p.?) 24%
TP53	DISRUPTIVE, SPLICE SITE	c.993+1G>T (p.?) 29%
TP53	DISRUPTIVE, SPLICE SITE	c.783-1G>T (p.?) 18%
TP53	DISRUPTIVE, SPLICE SITE	c.375G>T (p.Thr125=) 35%
TP53	DISRUPTIVE, SPLICE SITE	c.673-2A>T (p.?) 55%
TP53	DISRUPTIVE, SPLICE SITE	c.376-1G>A (p.?) 25%
TP53	DISRUPTIVE, SPLICE SITE	c.375G>T (p.Thr125=) 26%
TP53	DISRUPTIVE, SPLICE SITE	c.920-2A>G (p.?) 56%
TP53	DISRUPTIVE, SPLICE SITE	c.559+1G>T (p.?) 9%
TP53	DISRUPTIVE. SPLICE SITE	c.993+1G>A (p.?) 37%
TP53	NON-DISRUPTIVE, FUNCTIONAL	c.100C>A (p.Pro34Thr) 6%
TP53	NON-DISRUPTIVE, FUNCTIONAL	c.100C>A (p.Pro34Thr) 6%
TP53	NON-DISRUPTIVE, FUNCTIONAL	c.993G>T (p.Gln331His) 18%
TP53	NON-DISRUPTIVE, FUNCTIONAL	c.672G>T (p.Glu224Asp) 44%
TP53	NON-DISRUPTIVE, GAIN OF FUNCTION	c.733G>A (p.Gly245Ser) 59%
TP53	NON-DISRUPTIVE, GAIN OF FUNCTION	c.820G>T (p.Val274Phe) 21%
TP53	NON-DISRUPTIVE, GAIN OF FUNCTION	c.818G>A (p.Arg273His) 40%
TP53	NON-DISRUPTIVE, GAIN OF FUNCTION	c.538G>A (p.Glu180Lys)
TP53	NON-DISRUPTIVE, GAIN OF FUNCTION	c.734G>T (p. Gly245Val) 20%
TP53	NON-DISRUPTIVE, GAIN OF FUNCTION	c.832C>T (p.Pro278Ser) 5%

TP53	NON-DISRUPTIVE, GAIN OF FUNCTION	c.1096T>G (p.ser366Ala) 39%
TP53	NON-DISRUPTIVE, GAIN OF FUNCTION	c.524G>A (p.Arg175His) 33%
TP53	NON-DISRUPTIVE, GAIN OF FUNCTION	c.1039G>A (p.Ala347Thr) 19%
TP53	NON-DISRUPTIVE, GAIN OF FUNCTION	c.747G>T (p.Arg249Ser) 7%
TP53	NON-DISRUPTIVE, GAIN OF FUNCTION	c.524G>A (p.Arg175His) 45%
TP53	NON-DISRUPTIVE, GAIN OF FUNCTION	c.818G>A (p.Arg273His) 36%
TP53	NON-DISRUPTIVE, GAIN OF FUNCTION	c.524G>A (p.Arg175His) 74%
TP53	NON-DISRUPTIVE, GAIN OF FUNCTION	c.839G>A (p.Arg280Lys) 28%
TP53	NON-DISRUPTIVE, GAIN OF FUNCTION	c.817C>T (p.Arg273Cys) 32%
TP53	NON-DISRUPTIVE, GAIN OF FUNCTION	c.844C>T (p.Arg282Trp)
TP53	NON-DISRUPTIVE, GAIN OF FUNCTION	c.817C>T (p.Arg273Cys) 19%
TP53	NON-DISRUPTIVE, GAIN OF FUNCTION	c.569C>T (p.Pro190Leu) 58%
TP53	NON-DISRUPTIVE, GAIN OF FUNCTION	c. 487T>C (p.Tyr163His) 65%
TP53	NON-DISRUPTIVE, GAIN OF FUNCTION	c. 469G>T (p.Val157Phe) 30%
TP53	NON-DISRUPTIVE, GAIN OF FUNCTION	c.1001G>T (p.Gly334Val) 89%
TP53	NON-DISRUPTIVE, GAIN OF FUNCTION	c.747G>T (p.Arg249Ser) 27%
TP53	NON-DISRUPTIVE, GAIN OF FUNCTION	c.832C>T (p.Pro278Ser) 82%
TP53	NON-DISRUPTIVE, GAIN OF FUNCTION	c.818G>A (p.Arg273His) 18%
TP53	NON-DISRUPTIVE, GAIN OF FUNCTION	c.733G>A (p.Gly245Ser) 49%
TP53	NON-DISRUPTIVE, GAIN OF FUNCTION	c.839G>C (p.Arg280Thr) 48%
TP53	NON-DISRUPTIVE, GAIN OF FUNCTION	c.839G>C (p.Arg280Thr) 10%
TP53	NON-DISRUPTIVE, GAIN OF FUNCTION	c.818G>A (p.Arg273His) 10%
TP53	NON-DISRUPTIVE, GAIN OF FUNCTION	c.747G>T (p.Arg249Ser) 39%
TP53	NON-DISRUPTIVE, GAIN OF FUNCTION	c.524G>A (p.Arg175His) 5%
TP53	NON-DISRUPTIVE, GAIN OF FUNCTION	c.817C>T (p.Arg273Cys) 16%
TP53	NON-DISRUPTIVE, GAIN OF FUNCTION	c.832C>T (p.Pro278Ser) 96%
TP53	NON-DISRUPTIVE, GAIN OF FUNCTION	c. 730G>T (p.Gly244Cys) 26%
TP53	NON-DISRUPTIVE, GAIN OF FUNCTION	c.375+1G>T (p. ?) 11%
TP53	NON-DISRUPTIVE, GAIN OF FUNCTION	c.818G>A (p.Arg273His) 24%
TP53	NON-DISRUPTIVE, GAIN OF FUNCTION	c.524G>A (p.Arg175His) 11%
TP53	NON-DISRUPTIVE, GAIN OF FUNCTION	c.733G>A (p.Gly245Ser)
TP53	NON-DISRUPTIVE, GAIN OF FUNCTION	c.524G>A (p.Arg175His)
TP53	NON-DISRUPTIVE, GAIN OF FUNCTION	c. 413C>T (p.Ala138Val) 42%
TP53	NON-DISRUPTIVE, GAIN OF FUNCTION	c.747G>T (p.Arg249Ser) 49%
TP53	NON-DISRUPTIVE, GAIN OF FUNCTION	c.314G>A (p.Gly105Asp) 57%
TP53	NON-DISRUPTIVE, GAIN OF FUNCTION	c.733G>A (p.Gly245Ser) 13%
TP53	NON-DISRUPTIVE, GAIN OF FUNCTION	c.844C>T (p.Arg282Trp) 25%
TP53	NON-DISRUPTIVE, GAIN OF FUNCTION	c.818G>A (p.Arg273His) 40%
TP53	NON-DISRUPTIVE, GAIN OF FUNCTION	c.820G>T (p.Val274Phe) 58%
TP53	NON-DISRUPTIVE, GAIN OF FUNCTION	c.524G>A (p.Arg175His) 11%
TP53	NON-DISRUPTIVE, GAIN OF FUNCTION	c.800G>C (p.Arg267Pro) 11%
TP53	NON-DISRUPTIVE, GAIN OF FUNCTION	c.747G>T (p.Arg249Ser) 29%
TP53	NON-DISRUPTIVE, GAIN OF FUNCTION	c.818G>A (p.Arg273His) 20%
TP53	NON-DISRUPTIVE, GAIN OF FUNCTION	c.839G>C (p.Arg280Thr) 37%
TP53	NON-DISRUPTIVE, GAIN OF FUNCTION	c.104T>G (p.Leu348Trp) 84%
TP53	NON-DISRUPTIVE, GAIN OF FUNCTION	c.1001G>T (p.Gly334Val) 13%
TP53	NON-DISRUPTIVE, GAIN OF FUNCTION	c.842A>G (p.Asp281Gly) 14%

TP53	NON-DISRUPTIVE, GAIN OF FUNCTION	c.842A>G (p.Asp281Gly) 87%
TP53	NON-DISRUPTIVE, IN-FRAME DELETION	c.393_395delCAA (p.Asn131del) 15%
TP53	NON-DISRUPTIVE, MISSENSE	c.734G>C (p.Gly245Ala) 27%
TP53	NON-DISRUPTIVE, MISSENSE	c.79C>T (p.Pro27Ser) 28%
TP53	NON-DISRUPTIVE, NON-FUNCTIONAL	c.1010G>T (p.Arg337Leu) 13%
TP53	NON-DISRUPTIVE, NON-FUNCTIONAL	c.797G>T (p.Gly266Val) 38%
TP53	NON-DISRUPTIVE, NON-FUNCTIONAL	c.854A>T (p.Glu285Val)
TP53	NON-DISRUPTIVE, NON-FUNCTIONAL	c.536A>G (p.His179Arg) 39%
TP53	NON-DISRUPTIVE, NON-FUNCTIONAL	c.451C>T (p.Pro151Ser)
TP53	NON-DISRUPTIVE, NON-FUNCTIONAL	c.694A>T (p.Ile232Phe)
TP53	NON-DISRUPTIVE, NON-FUNCTIONAL	c.817C>T (p.Arg273Cis) 15%
TP53	NON-DISRUPTIVE, NON-FUNCTIONAL	c.488A>G (p.Tyr163Cys) 94%
TP53	NON-DISRUPTIVE, NON-FUNCTIONAL	c.772G>A (p.Glu258Lys)
TP53	NON-DISRUPTIVE, NON-FUNCTIONAL	c.583A>T (p.Ile195Phe) 61%
TP53	NON-DISRUPTIVE, NON-FUNCTIONAL	c.319T>C (p.Tyr107His) 25%
TP53	NON-DISRUPTIVE, NON-FUNCTIONAL	c.743G>T (p.Arg248Leu) 32%
TP53	NON-DISRUPTIVE, NON-FUNCTIONAL	c.794T>C (p.Leu265Pro) 11%
TP53	NON-DISRUPTIVE, NON-FUNCTIONAL	c.797G>T (p.Gly266Val) 17%
TP53	NON-DISRUPTIVE, NON-FUNCTIONAL	c.815T>G (p.Val272Gly)
TP53	NON-DISRUPTIVE, NON-FUNCTIONAL	c.722C>G (p.Ser241Cys) 16%
TP53	NON-DISRUPTIVE, NON-FUNCTIONAL	c.842A>C (p.Asp281Ala) 16%
TP53	NON-DISRUPTIVE, NON-FUNCTIONAL	c.772G>T (p.Glu258*) 32%
TP53	NON-DISRUPTIVE, NON-FUNCTIONAL	c.713G>A (p.Cys238Tyr) 25%
TP53	NON-DISRUPTIVE, NON-FUNCTIONAL	c.427G>A (p.Val143Met) 34%
TP53	NON-DISRUPTIVE, NON-FUNCTIONAL	c.536A>G (p.His179Arg) 13%
TP53	NON-DISRUPTIVE, NON-FUNCTIONAL	c.736A>G (p.Met246Val) 72%
TP53	NON-DISRUPTIVE, NON-FUNCTIONAL	c.818G>T (p.Arg273Leu) 23%
TP53	NON-DISRUPTIVE, NON-FUNCTIONAL	c.844C>T (p.Arg282Trp) 4%
TP53	NON-DISRUPTIVE, NON-FUNCTIONAL	c.995T>A (p.Ile332Asn) 37%
TP53	NON-DISRUPTIVE, NON-FUNCTIONAL	c.833C>G (p.Pro278Arg) 55%
TP53	NON-DISRUPTIVE, NON-FUNCTIONAL	c.488A>G (p.Tyr163Cys) 10%
TP53	NON-DISRUPTIVE, NON-FUNCTIONAL	c.475G>C (p.Ala159Pro) 10%
TP53	NON-DISRUPTIVE, NON-FUNCTIONAL	c.473G>T (p.Arg158Leu) 12%
TP53	NON-DISRUPTIVE, NON-FUNCTIONAL	c.461G>T (p.Gly154Val) 16%
TP53	NON-DISRUPTIVE, NON-FUNCTIONAL	c.469G>T (p.Val157Phe) 21%
TP53	NON-DISRUPTIVE, NON-FUNCTIONAL	c.839G>C (p. Arg280Thr) 50%
TP53	NON-DISRUPTIVE, NON-FUNCTIONAL	c.536A>G (p. His179Arg) 25%
TP53	NON-DISRUPTIVE, NON-FUNCTIONAL	c.701A>G (p.Tyr234Cys) 18%
TP53	NON-DISRUPTIVE, NON-FUNCTIONAL	c.473G>T (p.Arg158Leu) 48%
TP53	NON-DISRUPTIVE, NON-FUNCTIONAL	c.821T>G (p.Val274Gly) 42%
TP53	NON-DISRUPTIVE, NON-FUNCTIONAL	c.1081G>T (p.Gly361Trp) 44%
TP53	NON-DISRUPTIVE, NON-FUNCTIONAL	c.818G>C (p.Arg273Pro) 43%
TP53	NON-DISRUPTIVE, NON-FUNCTIONAL	c.404G>A (p.Cys135Tyr) 61%
TP53	NON-DISRUPTIVE, NON-FUNCTIONAL	c.536A>G (p.His179Arg) 40%
TP53	NON-DISRUPTIVE, NON-FUNCTIONAL	c.473G>T (p.Arg158Leu)
TP53	NON-DISRUPTIVE, NON-FUNCTIONAL	c.856G>A (p.Glu286Lys) 29%
TP53	NON-DISRUPTIVE, NON-FUNCTIONAL	c.644G>T (p.Ser215Ile)

TP53	NON-DISRUPTIVE, NON-FUNCTIONAL	c.473G>C (p.Arg158Pro) 13%
TP53	NON-DISRUPTIVE, NON-FUNCTIONAL	c.469G>T (p.Val157Phe) 13%
TP53	NON-DISRUPTIVE, NON-FUNCTIONAL	c.716A>G (p. Asn239Ser) 45%
TP53	NON-DISRUPTIVE, NON-FUNCTIONAL	c.817C>T (p. Arg273Cis) 25%
TP53	NON-DISRUPTIVE, NON-FUNCTIONAL	c.650T>G (p. Val217Gly) 20%
TP53	NON-DISRUPTIVE, NON-FUNCTIONAL	c.469G>T (p.Val157Phe) 75%
TP53	NON-DISRUPTIVE, NON-FUNCTIONAL	c.475G>C (p.Ala159Pro) 23%
TP53	NON-DISRUPTIVE, NON-FUNCTIONAL	c.734G>T (p.Gly245Val)
TP53	NON-DISRUPTIVE, NON-FUNCTIONAL	c.473G>T (p.Arg158Leu) 62%
TP53	NON-DISRUPTIVE, NON-FUNCTIONAL	c.838A>G (p.Arg280Gly) 17%
TP53	NON-DISRUPTIVE, NON-FUNCTIONAL	c.638G>T (p. Arg213Leu) 13%
TP53	NON-DISRUPTIVE, NON-FUNCTIONAL	c.454C>A (p.Pro152Thr) (60%)
TP53	NON-DISRUPTIVE, NON-FUNCTIONAL	c.841G>A (p.Asp281Asn) 9%
TP53	NON-DISRUPTIVE, NON-FUNCTIONAL	c.736A>G (p.Met246Val) 18%
TP53	NON-DISRUPTIVE, NON-FUNCTIONAL	c.797G>A (p.Gly266Glu) 50%
TP53	NON-DISRUPTIVE, NON-FUNCTIONAL	c.722C>G (p.Ser241Cys) 35%
TP53	NON-DISRUPTIVE, NON-FUNCTIONAL	c.469G>T (p.Val157Phe)
TP53	NON-DISRUPTIVE, NON-FUNCTIONAL	c.313G>T (p.Gly105Cys) 27%
TP53	NON-DISRUPTIVE, NON-FUNCTIONAL	c.469G>T (p.Val157Phe)
TP53	NON-DISRUPTIVE, NON-FUNCTIONAL	c.646G>A (p.Val216Met) 83%
TP53	NON-DISRUPTIVE, NON-FUNCTIONAL	c.814G>A (p.Val272Met) 49%
TP53	NON-DISRUPTIVE, NON-FUNCTIONAL	c.581T>G (p.Leu194Arg) 64%
TP53	NON-DISRUPTIVE, NON-FUNCTIONAL	c.476C>T (p.Ala159Val) 55%
TP53	NON-DISRUPTIVE, NON-FUNCTIONAL	c.818G>T (p.Arg273Leu) 31%
TP53	NON-DISRUPTIVE, NON-FUNCTIONAL	c.848G>C (p.Arg283Pro) (26%)
TP53	NON-DISRUPTIVE, NON-FUNCTIONAL	c.844C>T (p.Arg282Trp) 6%
TP53	NON-DISRUPTIVE, NON-FUNCTIONAL	c.404G>A (p.Cys135Tyr) 36%
TP53	NON-DISRUPTIVE, NON-FUNCTIONAL	c.313G>C (p.Gly105Arg) 15%
TP53	NON-DISRUPTIVE, NON-FUNCTIONAL	c.809T>C (p.Phe270Ser) 28%
TP53	NON-DISRUPTIVE, NON-FUNCTIONAL	c.818G>C (p.Arg273Pro) 82%
TP53	NON-DISRUPTIVE, NON-FUNCTIONAL	c.338T>G (p.Phe113Cys)
TP53	NON-DISRUPTIVE, NON-FUNCTIONAL	c.641A>G (p.His214Arg)
TP53	NON-DISRUPTIVE, NON-FUNCTIONAL	c.853G>A (p.Glu285Lys) 28%
TP53	NON-DISRUPTIVE, NON-FUNCTIONAL	c.473G>T (p.Arg158Leu) 12%
TP53	NON-DISRUPTIVE, NON-FUNCTIONAL	c.833C>T (p.Pro278Leu) 17%
TP53	NON-DISRUPTIVE, NON-FUNCTIONAL	c.830G>T (p.Cys277Phe) 22%
TP53	NON-DISRUPTIVE, NON-FUNCTIONAL	c.467G>C (p.Arg156Pro) 7%
TP53	NON-DISRUPTIVE, NON-FUNCTIONAL	c.374C>T (p.Thr125Met) 55%
TP53	NON-DISRUPTIVE, NON-FUNCTIONAL	c.396G>C (p.Lys132Asn) 19%
TP53	NON-DISRUPTIVE, NON-FUNCTIONAL	c.581T>G (p.Leu194Arg) 37%
TP53	NON-DISRUPTIVE, NON-FUNCTIONAL	c.581T>G (p.Leu194Arg) 41%
TP53	NON-DISRUPTIVE, NON-FUNCTIONAL	c.1009C>T (p.Arg337Cys) 30%
TP53	NON-DISRUPTIVE, NON-FUNCTIONAL	c.794T>G (p.Leu265Arg) 18%
TP53	NON-DISRUPTIVE, NON-FUNCTIONAL	c.646G>T (p.Val216Leu) 8%
TP53	NON-DISRUPTIVE, NON-FUNCTIONAL	c.820G>T (p.Val274Phe) 21%
TP53	NON-DISRUPTIVE, NON-FUNCTIONAL	c.731G>T (p.Gly244Val) 88%
TP53	NON-DISRUPTIVE, NON-FUNCTIONAL	c.818G>T (p.Arg273Leu) 48%

TP53	NON-DISRUPTIVE, NON-FUNCTIONAL	c.818G>C (p.Arg273Pro) 28%
TP53	NON-DISRUPTIVE, NON-FUNCTIONAL	c.580C>T (p.Leu194Phe) 9%
TP53	NON-DISRUPTIVE, NON-FUNCTIONAL	c.731G>T (p.Gly244Val) 12%
TP53	NON-DISRUPTIVE, NON-FUNCTIONAL	c.830G>T (p.Cys277Phe) 15%
TP53	NON-DISRUPTIVE, NON-FUNCTIONAL	c.848G>C (p.Arg283Pro) 46%
TP53	NON-DISRUPTIVE, NON-FUNCTIONAL	c.473G>T (p.Arg158Leu) 35%
TP53	NON-DISRUPTIVE, NON-FUNCTIONAL	c.646G>A (p.Val216Met) 12%
TP53	NON-DISRUPTIVE, NON-FUNCTIONAL	c.503A>G (p.His168Arg) 18%
TP53	NON-DISRUPTIVE, NON-FUNCTIONAL	c.578T>G (p.His193Arg) 30%
TP53	NON-DISRUPTIVE, NON-FUNCTIONAL	c.1010G>T (p.Arg337Leu) 97%
TP53	NON-DISRUPTIVE, NON-FUNCTIONAL	c.517G>A (p.Val173Met) 43%
TP53	NON-DISRUPTIVE, NON-FUNCTIONAL	c.743G>T (p.Arg248Leu)
TP53	NON-DISRUPTIVE, NON-FUNCTIONAL	c.484A>T (p.Ile162Phe) 7%
TP53	NON-DISRUPTIVE, NON-FUNCTIONAL	c.796G>A (p.Gly266Arg) 30%
TP53	NON-DISRUPTIVE, NON-FUNCTIONAL	c.743G>T (p.Arg248Leu) 8%
TP53	NON-DISRUPTIVE, NON-FUNCTIONAL	c.614A>G (p.Tyr205Cys)
TP53	NON-DISRUPTIVE, NON-FUNCTIONAL	c.848G>C (p. Arg283Pro) 47%
TP53	NON-DISRUPTIVE, NON-FUNCTIONAL	c.658T>C (p.Tyr220His) 26%
TP53	NON-DISRUPTIVE, NON-FUNCTIONAL	c.332T>C (p.Leu111Pro) 28%
TP53	NON-DISRUPTIVE, NON-FUNCTIONAL	c.843C>G (p.Asp281Glu) 5%
TP53	NON-DISRUPTIVE, NON-FUNCTIONAL	c.812A>T (p.Glu271Val) 6%
TP53	NON-DISRUPTIVE, NON-FUNCTIONAL	c.818G>T (p.Arg273Leu) 6%
TP53	NON-DISRUPTIVE, NON-FUNCTIONAL	c.514G>T (p.Val172Phe) 26%
TP53	NON-DISRUPTIVE, NON-FUNCTIONAL	c.830G>T (p.Cys277Phe) 44%
TP53	NON-DISRUPTIVE, NON-FUNCTIONAL	c.473G>T (p.Arg158Leu) 24%
TP53	NON-DISRUPTIVE, NON-FUNCTIONAL	c.527G>A (p.Cys176Tyr) 34%
TP53	NON-DISRUPTIVE, NON-FUNCTIONAL	c.818G>T (p.Arg273Leu) 12%
TP53	NON-DISRUPTIVE, NON-FUNCTIONAL	c.638G>A (p.Arg213Gln) 5%
TP53	NON-DISRUPTIVE, NON-FUNCTIONAL	c.469G>T (p. Val157Phe) 27%
TP53	NON-DISRUPTIVE, NON-FUNCTIONAL	c.722C>A (p.Ser241Tyr) 26%
TP53	NON-DISRUPTIVE, NON-FUNCTIONAL	c.770_779delinsCGGAAGACTT (p.Leu257_Ser260delinsProGluAspPhe) 10%
TP53	NON-DISRUPTIVE, NON-FUNCTIONAL	c.614A>G (p.Tyr205Cys) 84%
TP53	NON-DISRUPTIVE, NON-FUNCTIONAL	c.475G>C (p.Ala159Pro) 17%
TP53	NON-DISRUPTIVE, NON-FUNCTIONAL	c.328C>T (p.Arg110Cys) 8%
TP53	NON-DISRUPTIVE, NON-FUNCTIONAL	c.859G>T (p.Glu287*) 12%
TP53	NON-DISRUPTIVE, NON-FUNCTIONAL	c.707A>G (p.Tyr236Cys) (45%)
TP53	NON-DISRUPTIVE, NON-FUNCTIONAL	c.824G>T (p.Cys275Phe) 24%
TP53	NON-DISRUPTIVE, NON-FUNCTIONAL	c.853G>A (p.Glu285Lys) 86%
TP53	NON-DISRUPTIVE, NON-FUNCTIONAL	c.517G>T (p.Val173Leu) 89%
TP53	NON-DISRUPTIVE, NON-FUNCTIONAL	c.517G>C (p.Val173Leu) 26%
TP53	NON-DISRUPTIVE, NON-FUNCTIONAL	c.517G>C (p.Val173Leu) 8%
TP53	NON-DISRUPTIVE, NON-FUNCTIONAL	c.743G>T (p.Arg248Leu) 10%
TP53	NON-DISRUPTIVE, NON-FUNCTIONAL	c.814G>T (p.Val272Leu) 14%
TP53	NON-DISRUPTIVE, NON-FUNCTIONAL	c.812A>T (p.Glu271Val) 41%
TP53	NON-DISRUPTIVE, NON-FUNCTIONAL	c.818G>T (p.Arg273Leu) 30%
TP53	NON-DISRUPTIVE, NON-FUNCTIONAL	c.701A>G (p.Tyr234Cys)
TP53	NON-DISRUPTIVE, NON-FUNCTIONAL	c.832C>A (p.Pro278Thr) 47%

TP53	NON-DISRUPTIVE, NON-FUNCTIONAL	c.482C>A (p.Ala161Asp) 7%
TP53	NON-DISRUPTIVE, NON-FUNCTIONAL	c.743G>T (p. Arg248Leu) 26%
TP53	NON-DISRUPTIVE, NON-FUNCTIONAL	c.398T>A (p.Met133Lys) 17%
TP53	NON-DISRUPTIVE, NON-FUNCTIONAL	c.398T>A (p.Met133Lys) 37%
TP53	NON-DISRUPTIVE, NON-FUNCTIONAL	c.817C>T (p.Arg273Cys) 71%
TP53	NON-DISRUPTIVE, NON-FUNCTIONAL	c.536A>G (p.His179Arg)
TP53	NON-DISRUPTIVE, NON-FUNCTIONAL	c.853G>A (p.Glu285Lys) 19%
TP53	NON-DISRUPTIVE, NON-FUNCTIONAL	c.730G>A (p.Gly244Ser) 43%
TP53	NON-DISRUPTIVE, NON-FUNCTIONAL	c.461G>T (p.Gly154Val) 23%
TP53	NON-DISRUPTIVE, NON-FUNCTIONAL	c.517G>C (p.Val173Leu) 49%
TP53	NON-DISRUPTIVE, NON-FUNCTIONAL	c.814G>T (p.Val272Leu) 9%
TP53	NON-DISRUPTIVE, NON-FUNCTIONAL	c.470T>G (p.Val157Gly) 56%
TP53	NON-DISRUPTIVE, NON-FUNCTIONAL	c.473G>T (p.Arg158Leu) 38%
TP53	NON-DISRUPTIVE, NON-FUNCTIONAL	c.469G>T (p.Val157Phe) 21%
TP53	NON-DISRUPTIVE, NON-FUNCTIONAL	c.797G>T (p.Gly266Val) 7%
TP53	NON-DISRUPTIVE, NON-FUNCTIONAL	c.853G>A (p.Glu285Lys) 56%
TP53	NON-DISRUPTIVE, NON-FUNCTIONAL	c.461G>T (p.Gly154Val)
TP53	NON-DISRUPTIVE, NON-FUNCTIONAL	c.455C>T (p. Pro152Leu) 33%
TP53	NON-DISRUPTIVE, NON-FUNCTIONAL	c.473G>C (p.Arg158Pro) 48%
TP53	NON-DISRUPTIVE, NON-FUNCTIONAL	c.832C>A (p.Pro278Thr) 36%
TP53	NON-DISRUPTIVE, NON-FUNCTIONAL	c.451C>A (p. Pro151Thr) 46%
TP53	NON-DISRUPTIVE, NON-FUNCTIONAL	c.823T>C (p.Cys275Arg) 34%
TP53	NON-DISRUPTIVE, NON-FUNCTIONAL	c.473G>T (p.Arg158Leu) 7%
TP53	NON-DISRUPTIVE, NON-FUNCTIONAL	c.380C>T (p.Ser127Phe) 78%
TP53	NON-DISRUPTIVE, NON-FUNCTIONAL	c.818G>T (p.Arg273Leu) 39%
TP53	NON-DISRUPTIVE, NON-FUNCTIONAL	c.473G>T (p.Arg158Leu) 46%
TP53	NON-DISRUPTIVE, NON-FUNCTIONAL	c.581T>G (p.Leu194Arg) 73%
TP53	NON-DISRUPTIVE, NON-FUNCTIONAL	c.106_108delCCG (p.Pro36del) 10%
TP53	NON-DISRUPTIVE, NON-FUNCTIONAL	c.817C>A (p.Arg273Ser) 26%
TP53	NON-DISRUPTIVE, NON-FUNCTIONAL	c.461_462delGCinsTT (p. Gly154Val) 26%
TP53	NON-DISRUPTIVE, NON-FUNCTIONAL	c.817C>A (p.Arg273Ser) 34%
TP53	NON-DISRUPTIVE, NON-FUNCTIONAL	c.808T>C (p.Phe270Leu) 6%
TP53	NON-DISRUPTIVE, NON-FUNCTIONAL	c.469G>T (p.Val157Phe) (31%)
TP53	NON-DISRUPTIVE, NON-FUNCTIONAL	c.578A>G (p.His193Arg) 20%
TP53	NON-DISRUPTIVE, NON-FUNCTIONAL	c.473G>T (p.Arg158Leu) 59%
TP53	NON-DISRUPTIVE, NON-FUNCTIONAL	c.581T>A (p.Leu194His) 16%
TP53	NON-DISRUPTIVE, NON-FUNCTIONAL	c.412G>C (p.Ala138Pro) 23%
TP53	NON-DISRUPTIVE, NON-FUNCTIONAL	c.1031T>G (p.Leu344Arg)
TP53	NON-DISRUPTIVE, NON-FUNCTIONAL	c.583A>T (p.Ile195Phe) 43%
TP53	NON-DISRUPTIVE, NON-FUNCTIONAL	c.853G>A (p.Glu285Lys) 27%
TP53	NON-DISRUPTIVE, NON-FUNCTIONAL	c.818G>T (p.Arg273Leu) 58%
TP53	NON-DISRUPTIVE, NON-FUNCTIONAL	c.379T>C (p.Ser127Pro) 57%
TP53	NON-DISRUPTIVE, NON-FUNCTIONAL	c.641A>G (p.His214Arg) 15%
TP53	NON-DISRUPTIVE, NON-FUNCTIONAL	c.817C>T (p.Arg273Cys)
TP53	NON-DISRUPTIVE, NON-FUNCTIONAL	c.380C>T (p.Ser127Phe) 24%
TP53	NON-DISRUPTIVE, NON-FUNCTIONAL	c.711G>C (p.Met237Ile) 34%
TP53	NON-DISRUPTIVE, NON-FUNCTIONAL	c.469G>T (p.Val157Phe) 14%

TP53	NON-DISRUPTIVE, NON-FUNCTIONAL	c.773A>C (p.Glu258Ala)
TP53	NON-DISRUPTIVE, NON-FUNCTIONAL	c.716A>G (p.Asn239Ser) 21%
TP53	NON-DISRUPTIVE, NON-FUNCTIONAL	c.725G>A (p.Cys242Tyr) 23%
TP53	NON-DISRUPTIVE, NON-FUNCTIONAL	c.514G>T (p.Val172Phe) 43%
TP53	NON-DISRUPTIVE, NON-FUNCTIONAL	c.707A>G (p.Tyr236Cys)
TP53	NON-DISRUPTIVE, NON-FUNCTIONAL	c.469G>T (p.Val157Phe)
TP53	NON-DISRUPTIVE, NON-FUNCTIONAL	c.772G>A (p.Glu258Lys)
TP53	NON-DISRUPTIVE, NON-FUNCTIONAL	c.488A>G (p.Tyr163Cys) 14%
TP53	NON-DISRUPTIVE, NON-FUNCTIONAL	c.581T>G (p.Leu194Arg)
TP53	NON-DISRUPTIVE, NON-FUNCTIONAL	c.423C>G (p.Cys141Trp) 51%
TP53	NON-DISRUPTIVE, NON-FUNCTIONAL	c.469G>T (p.Val157Phe)
TP53	NON-DISRUPTIVE, NON-FUNCTIONAL	c.659A>G (p.Tyr220Cys) 13%
TP53	NON-DISRUPTIVE, NON-FUNCTIONAL	c.734G>T (p.Gly245Val)
TP53	NON-DISRUPTIVE, NON-FUNCTIONAL	c.725G>C (p.Cys242Ser) 64%
TP53	NON-DISRUPTIVE, NON-FUNCTIONAL	c.857A>T (p.Glu286Val)
TP53	NON-DISRUPTIVE, NON-FUNCTIONAL	c.461G>T (p.Gly154Val) 33%
TP53	NON-DISRUPTIVE, NON-FUNCTIONAL	c.347C>G (p.Ser116Cys) 81%
TP53	NON-DISRUPTIVE, NON-FUNCTIONAL	c.469G>T (p.Val157Phe) 20%
TP53	NON-DISRUPTIVE, NON-FUNCTIONAL	c.659A>G (p.Tyr220Cys) 13%
TP53	NON-DISRUPTIVE, NON-FUNCTIONAL	c.464C>A (p.Thr155Asn) 64%
TP53	NON-DISRUPTIVE, NON-FUNCTIONAL	c.995T>G (p.Ile332Ser) 31%
TP53	NON-DISRUPTIVE, NON-FUNCTIONAL	c.814G>C (p.Val272Leu) 43%
TP53	NON-DISRUPTIVE, NON-FUNCTIONAL	c.396G>T (p.Lys132Asn) 63%
TP53	NON-DISRUPTIVE, NON-FUNCTIONAL	c.374C>G (p.Thr125Arg) 35%
TP53	NON-DISRUPTIVE, NON-FUNCTIONAL	c.707A>G (p.Tyr236Cys) 18%
TP53	NON-DISRUPTIVE, NON-FUNCTIONAL	c.738G>T (p.Met246Ile) 15%
TP53	NON-DISRUPTIVE, NON-FUNCTIONAL	c.644G>T (p.Ser215Ile) 76%
TP53	NON-DISRUPTIVE, NON-FUNCTIONAL	c.796G>A (p.Gly266Arg) 85%
TP53	NON-DISRUPTIVE, NON-FUNCTIONAL	c.818G>T (p.Arg273Leu) 33%
TP53	NON-DISRUPTIVE, NON-FUNCTIONAL	c.470T>A (p.Val157Asp) 12%
TP53	NON-DISRUPTIVE, NON-FUNCTIONAL	c.473G>T (p.Arg158Leu) 31%
TP53	NON-DISRUPTIVE, NON-FUNCTIONAL	c.461G>T (p.Gly154Val) 74%
TP53	NON-DISRUPTIVE, NON-FUNCTIONAL	c.646G>A (p.Val216Met) 14%
TP53	NON-DISRUPTIVE, NON-FUNCTIONAL	c.856G>C (p.Glu286Gln) 80%
TP53	NON-DISRUPTIVE, NON-FUNCTIONAL	c.461G>T (p.Gly154Val) 32%
TP53	NON-DISRUPTIVE, NON-FUNCTIONAL	c.329G>C (p.Arg110Pro) 30%
TP53	NON-DISRUPTIVE, NON-FUNCTIONAL	c.423C>G (p.Cys141Trp) 13%
TP53	NON-DISRUPTIVE, NON-FUNCTIONAL	c.830G>T (p.Cys277Phe) 35%
TP53	NON-DISRUPTIVE, NON-FUNCTIONAL	c.398T>C (p.Met133Thr) 34%
TP53	NON-DISRUPTIVE, NON-FUNCTIONAL	c.643A>G (p.Ser215Gly) 26%
TP53	NON-DISRUPTIVE, NON-FUNCTIONAL	c.711G>A (p.Met237Ile) 63%
TP53	NON-DISRUPTIVE, NON-FUNCTIONAL	c.830G>T (p.Cys277Phe) 25%
TP53	NON-DISRUPTIVE, NON-FUNCTIONAL	c.700T>C (p.Tyr234His) 94%
TP53	NON-DISRUPTIVE, NON-FUNCTIONAL	c.473G>C (p.Arg158Pro) 30%
TP53	NON-DISRUPTIVE, NON-FUNCTIONAL	c.707A>G (p.Tyr236Cys) 75%
TP53	NON-DISRUPTIVE, NON-FUNCTIONAL	c.761T>C (p.Ile254Thr) 6%
TP53	NON-DISRUPTIVE, NON-FUNCTIONAL	c.469G>T (p.Val157Phe) 18%

TP53	NON-DISRUPTIVE, NON-FUNCTIONAL	c.514G>T (p.Val172Phe) 77%
TP53	NON-DISRUPTIVE, NON-FUNCTIONAL	c.514G>T (p.Val172Phe) 16%
TP53	NON-DISRUPTIVE, NON-FUNCTIONAL	c.824G>T (p.Cys275Phe) 19%
TP53	NON-DISRUPTIVE, NON-FUNCTIONAL	c.818G>T (p.Arg273Leu) 25%
TP53	NON-DISRUPTIVE, NON-FUNCTIONAL	c.461G>T (p.Gly154Val) 35%
TP53	NON-DISRUPTIVE, NON-FUNCTIONAL	c.1009C>T (p.Arg337Cys) 46%
TP53	NON-DISRUPTIVE, NON-FUNCTIONAL	c.391A>T (p.Asn131Tyr) 29%
TP53	NON-DISRUPTIVE, NON-FUNCTIONAL	c.818G>T (p.Arg273Leu) 14%
TP53	NON-DISRUPTIVE, NON-FUNCTIONAL	c.517G>T (p.Val173Leu) 39%
TP53	NON-DISRUPTIVE, NON-FUNCTIONAL	c.818G>T (p.Arg273Leu) 10%
TP53	NON-DISRUPTIVE, NON-FUNCTIONAL	c.1010G>T (p.Arg337Leu) 28%
TP53	NON-DISRUPTIVE, NON-FUNCTIONAL	c.722C>A (p.Ser241Tyr) 62%
TP53	NON-DISRUPTIVE, NON-FUNCTIONAL	c.380C>T (p.Ser127Phe) 82%
TP53	NON-DISRUPTIVE, NON-FUNCTIONAL	c.473G>T (p.Arg158Leu) 80%
TP53	NON-DISRUPTIVE, NON-FUNCTIONAL	c.469G>T (p.Val157Phe) 46%
TP53	NON-DISRUPTIVE, NON-FUNCTIONAL	c.464C>T (p.Thr155Ile) 32%
TP53	NON-DISRUPTIVE, NON-FUNCTIONAL	c.644G>T (p.Ser215Ile) 24%
TP53	NON-DISRUPTIVE, NON-FUNCTIONAL	c.736A>T (p.Met246Leu) 17%
TP53	NON-DISRUPTIVE, NON-FUNCTIONAL	c.701A>G (p.Tyr234Cys) 28%
TP53	NON-DISRUPTIVE, NON-FUNCTIONAL	c.853G>A (p.Glu285Lys) 46%
TP53	NON-DISRUPTIVE, NON-FUNCTIONAL	c.517G>A (p.Val173Met) 64%
TP53	NON-DISRUPTIVE, NON-FUNCTIONAL	c.329G>C (p.Arg110Pro) 25%
TP53	NON-DISRUPTIVE, NON-FUNCTIONAL	c.578A>G (p.His193Arg) 25%
TP53	NON-DISRUPTIVE, NON-FUNCTIONAL	c.461G>T (p.Gly154Val) 13%
TP53	NON-DISRUPTIVE, NON-FUNCTIONAL	c.646G>T (p.Val216Leu) 42%
TP53	NON-DISRUPTIVE, NON-FUNCTIONAL	c.711G>A (p.Met237Ile) 17%
TP53	NON-DISRUPTIVE, NON-FUNCTIONAL	c.376T>A (p.Tyr126Asn) (90%
TP53	NON-DISRUPTIVE, NON-FUNCTIONAL	c.396G>C (p.Lys132Asn) 13%
TP53	NON-DISRUPTIVE, NON-FUNCTIONAL	c.701A>G (p.Tyr234Cys) 5%
TP53	NON-DISRUPTIVE, NON-FUNCTIONAL	c.313G>T (p.Gly105Cys) 67%
TP53	NON-DISRUPTIVE, NON-FUNCTIONAL	c.818G>T (p.Arg273Leu) 21%
TP53	NON-DISRUPTIVE, NON-FUNCTIONAL	c.638G>A (p.Arg213Gln) 60%
TP53	NON-DISRUPTIVE, NON-FUNCTIONAL	c.380C>A (p. Ser127Tyr) 15%
TP53	NON-DISRUPTIVE, NON-FUNCTIONAL	c.853G>A (p.Glu285Lys) 38%
TP53	NON-DISRUPTIVE, NON-FUNCTIONAL	c.814G>T (p.Val272Leu) 29%
TP53	NON-DISRUPTIVE, NON-FUNCTIONAL	c.578A>G (p.His193Arg) 68%
TP53	NON-DISRUPTIVE, NON-FUNCTIONAL	c.995T>A (p.Ile332Asn) 11%
TP53	NON-DISRUPTIVE, NON-FUNCTIONAL	c.824G>T (p.Cys275Phe) 10%
TP53	NON-DISRUPTIVE, NON-FUNCTIONAL	c.736A>T (p.Met246Leu) 26%
TP53	NON-DISRUPTIVE, NON-FUNCTIONAL	c.454C>T (p.Pro152Ser) 25%
TP53	NON-DISRUPTIVE, NON-SENSE	c.139C>T (p.Pro47Ser) 97%
TP53	NON-DISRUPTIVE, NOS	c.646_651 delGTGGTGinsTTGGGT (p.Val216_Val217delinsLeuGly) 52%
TP53	NON-DISRUPTIVE, NOS	c.530C>T (p.Pro177Leu) 28%
TP53	NON-DISRUPTIVE, NOS	c.844C>G (p.Arg282Gly) 25%
TP53	NON-DISRUPTIVE, NOS	c.659A>G (p.Tyr220Cys) 13%
TP53	NON-DISRUPTIVE, NOS	c.797G>A (p. Gly266Glu) 63%
TP53	NON-DISRUPTIVE, NOS	c.947C>T (p.Pro316Leu) 48%

TP53	NON-DISRUPTIVE, NOS	c.329G>T (p.Arg110Leu) 49%
TP53	NON-DISRUPTIVE, NOS	c.659A>G (p. Tyr220Cys) 28%
TP53	NON-DISRUPTIVE, NOS	c.450_455delACCCCC (p.Pro152_Pro153del) 54%
TP53	NON-DISRUPTIVE, NOS	c.772G>C (p.Glu258Gln) 21%
TP53	NON-DISRUPTIVE, NOS	c.127T>G (p.Leu43Val) 32%
TP53	NON-DISRUPTIVE, NOS	c.659A>G (p.Tyr220Cys) 13%
TP53	NON-DISRUPTIVE, NOS	c.826G>T (p.Ala276Ser) 13%
TP53	NON-DISRUPTIVE, NOS	c.614A>T (p.Tyr205Phe) 12%
TP53	NON-DISRUPTIVE, NOS	c.476C>T (p.Ala159Val) 15%
TP53	NON-DISRUPTIVE, NOS	c.480_481delGGinsTT (p.Met160_Ala161delinsIleSer) 20%
TP53	NON-DISRUPTIVE, NOS	c.457_459delCCC (p.Pro153del)
TP53	NON-DISRUPTIVE, NOS	c.841G>C (p.Asp281His) 58%
TP53	NON-DISRUPTIVE, NOS	c.404G>T (p.Cys135Phe) 28%
TP53	NON-DISRUPTIVE, NOS	c.419_427delCCTGCCCTG (p.Thr140_Val143delinsMet) 26%
TP53	NON-DISRUPTIVE, NOS	c.973G>A (p.Gly325Arg) 25%
TP53	NON-DISRUPTIVE, NOS	c.376_381delTACTCC (p.Tyr126_Ser127del) 31%
TP53	NON-DISRUPTIVE, NOS	c.473G>T (p. Arg158Leu) 32%
TP53	NON-DISRUPTIVE, NOS	c.730G>T (p.Gly244Cys) 15%
TP53	NON-DISRUPTIVE, NOS	c.812_826delAGGTGCGTGTTTGTG (p.Glu271_Cys275del) 20%
TP53	NON-DISRUPTIVE, NOS	c.401_406dupTTTGCC (p.Cys135_Gln136insLeuCys) 16%
TP53	NON-DISRUPTIVE, NOS	c.756_770delCACCATCATCACACT (p.Thr253_Leu257del)
TP53	NON-DISRUPTIVE, NOS	c.994-1G>A (p.?) 36%
TP53	NON-DISRUPTIVE, NOS	c.91G>A (p. Val31Ile) 30%
TP53	NON-DISRUPTIVE, NOS	c.841G>C (p.Asp281His) 25%
TP53	NON-DISRUPTIVE, NOS	c.393_395delCAA (p.Asn131del) 42%
TP53	NON-DISRUPTIVE, NOS	c.376-2A>G (p. ?) 42%
TP53	NON-DISRUPTIVE, NOS	c.818G>A (p.Arg273His) 44%
TP53	NON-DISRUPTIVE, NOS	c.659A>G (p.Tyr220Cys) 31%
TP53	NON-DISRUPTIVE, NOS	c.856G>A (p.Glu286Lys) 26%
TP53	NON-DISRUPTIVE, NOS	c.383_391delCTGCCCTCA (p. Pro128_Asn131delinsHis) 10%
TP53	NON-DISRUPTIVE, NOS	c.659A>G (p.Tyr220Cys) 68%
TP53	NON-DISRUPTIVE, NOS	c.839G>A (p.Arg280Lys) 43%
TP53	NON-DISRUPTIVE, NOS	c.747G>C (p.Arg249Ser) 12%
TP53	NON-DISRUPTIVE, NOS	c.586C>G (p.Arg196Gly) 26%
TP53	NON-DISRUPTIVE, NOS	c.797G>A (p.Gly266Glu) 50%
TP53	NON-DISRUPTIVE, NOS	c.476C>T (p.Ala159Val)
TP53	NON-DISRUPTIVE, NOS	c.476C>T (p.Ala159Val) 30%
TP53	NON-DISRUPTIVE, NOS	c.659A>G (p.Tyr220Cys) 71%
TP53	NON-DISRUPTIVE, NOS	c.404G>T (p.Cys135Phe)
TP53	NON-DISRUPTIVE, NOS	c.329G>T (p.Arg110Leu) 18%
TP53	NON-DISRUPTIVE, NOS	c.467G>C (p.Arg156Pro) 23%
TP53	NON-DISRUPTIVE, NOS	c.839G>A (p.Arg280Lys)
TP53	NON-DISRUPTIVE, NOS	c.659A>G (p. Tyr220Cys) 39%
TP53	NON-DISRUPTIVE, NOS	c.847C>T (p.Arg283Cys) 57%
TP53	NON-DISRUPTIVE, NOS	c.1053_1054delinsCT (p.Lys351_Asp352delinsAsnTyr) 30%
TP53	NON-DISRUPTIVE, NOS	c.393_395delCAA (p.Asn131del)
TP53	NON-DISRUPTIVE, NOS	c.773A>C (p.Glu258Ala) 33%

TP53	NON-DISRUPTIVE, NOS	c.1001_1006delGGCGTG (p.Gly334_Arg335del) 85%
TP53	NON-DISRUPTIVE, NOS	c.622G>A (p.Asp208Asn) 15%
TP53	NON-DISRUPTIVE, NOS	c.329G>T (p.Arg110Leu) 36%
TP53	NON-DISRUPTIVE, NOS	c.842A>T (p.Asp281Val) 69%
TP53	NON-DISRUPTIVE, NOS	c.329G>T (p.Arg110Leu) 80%
TP53	NON-DISRUPTIVE, NOS	c.821_826delTTTGTG (p. Val274_Cys275del) 36%
TP53	NON-DISRUPTIVE, NOS	c.659A>G (p.Tyr220Cys) 21%
TP53	NON-DISRUPTIVE, PARTIALLY FUNCTIONAL	c.640C>T (p.His214Tyr) 61%
TP53	NON-DISRUPTIVE, PARTIALLY FUNCTIONAL	c.542G>A (p.Arg181His) (10%)
TP53	NON-DISRUPTIVE, PARTIALLY FUNCTIONAL	c.587G>A (p.Arg196Gln) 20%
TP53	NON-DISRUPTIVE, PARTIALLY FUNCTIONAL	c.1010G>A (p.Arg337His) 11%
TP53	NON-DISRUPTIVE, PARTIALLY FUNCTIONAL	c.480G>A (p.Met160Ile) 72%
TP53	NON-DISRUPTIVE, PARTIALLY FUNCTIONAL	c.569C>T (p.Pro190Leu) 12%
TP53	NON-DISRUPTIVE, PARTIALLY FUNCTIONAL	c.405C>G (p.Cys135Trp) 10%
TP53	NON-DISRUPTIVE, PARTIALLY FUNCTIONAL	c.608T>A (p.Val203Glu) 39%
TP53	NON-DISRUPTIVE, PARTIALLY FUNCTIONAL	c.607G>T (p.Val203Leu) 56%
TP53	NON-DISRUPTIVE, PARTIALLY FUNCTIONAL	c.569C>T (p.Pro190Leu) 9%
TP53	NON-DISRUPTIVE, PARTIALLY FUNCTIONAL	c.166G>A (p.Glu56Lys) 46%
TP53	NON-DISRUPTIVE, PARTIALLY FUNCTIONAL	c.413C>T (p.Ala138Val) 24%
TP53	NON-DISRUPTIVE, PARTIALLY FUNCTIONAL	c.413C>T (p.ALa138Val) 5%
TP53	NON-DISRUPTIVE, PARTIALLY FUNCTIONAL	c.587G>A (p.Arg196Gln) 37%
TP53	NON-FUNCTIONAL	c.742C>T (p.Arg248Trp) 5%
TP53	UNCLEAR	c.532delC (p.His178Thrfs*69) 24%
TP53	UNCLEAR	c.994-1G>C (p.?) 64%
TP53	UNCLEAR	c.376-13_376delCCTCTTCCTACAGT (p.) 23%
TP53	UNCLEAR	c.903delA (p.Ser303Alafs*42) 6%
TP53	UNCLEAR	c.321C>G (p.Tyr107*) 15%
TP53	UNCLEAR	c.811G>T (p.Glu271*) 11%
TP53	UNCLEAR	c.818G>T (p.Arg273Leu) 15%
